# AI Adoption, Adaptation, and Workforce Sustainability in Medical Imaging: Systematic Review and Conceptual Framework

**DOI:** 10.2196/101944

**Published:** 2026-11-03

**Authors:** Paula Brough, Khondker Mohammad Zobair, Javad Pool, Natalie Haskell, Luke Houghton, Paul Scuffham, Jennifer Loy, Ashlea Troth, Salim Memon, Rachael Smithson, Maria Khan, Brooke Murphy, Letisha Living, Leanne Du, John Richards, Jennifer Dolkens, Matthew Aitken, Jason Zhang, John Grieve, Sandeep Bhuta

**Affiliations:** 1 Centre for Work, Organisation and Wellbeing, Griffith University, 170 Kessels Road, Nathan, Brisbane, Queensland, 4111, Australia, 61 0737354856; 2 Griffith University, Gold Coast, Queensland, Australia; 3 School of Medicine and Dentistry, Griffith University, Gold Coast, Australia; 4 Gold Coast Health, Gold Coast, Australia; 5 QUT Business School, Queensland University of Technology, Brisbane, Australia; 6 Gold Coast University Hospital, Gold Coast Health, Gold Coast, Australia

**Keywords:** medical imaging, radiology, workforce sustainability, AI adoption, AI adaptation, adaptation

## Abstract

**Background:**

Despite growing recognition that AI-driven systems offer transformative benefits for diagnostic imaging, insufficient evidence exists regarding how medical imaging workforces adapt to AI technology to support workforce sustainability.

**Objective:**

The aim of this study is to systematically review the existing literature on perceptions and attitudes toward AI adoption in medical imaging, postadoption adaptation behaviors within the workforce, and the implications of these processes for workforce sustainability.

**Methods:**

Following the PRISMA (Preferred Reporting Items for Systematic Reviews and Meta-Analyses) guidelines, a systematic search was carried out across 7 databases. Thematic analysis was used to systematically identify, analyze, and interpret patterns across the literature, providing a comprehensive understanding of workforce perceptions and adaptation behaviors.

**Results:**

A total of 69 peer-reviewed articles were included for thematic synthesis. The findings produced 6 critical factors that were identified as themes associated with AI adoption: perceived benefits, trust, readiness to change, autonomy and control, perceived threat to professional identity, and attitudes and intentions toward AI adoption and use. Four key facets of effective cognitive and behavioral adaptation were identified: training and skills development, task performance during AI-assisted practice, reduction of mental health burden and work strain, and policy compliance. The influence of work engagement and job satisfaction on workforce sustainability was also conceptualized. The findings were integrated into a framework describing the radiology workforce perspectives on AI adoption, adaptation behaviors, and workforce sustainability.

**Conclusions:**

The findings have potential implications for policy, particularly in strengthening AI-equipped medical imaging systems, fostering trust in AI-driven technologies, and supporting workforce training and upskilling, while considering professional autonomy and role identity. The review highlights a research gap, emphasizing the need for further investigation into postadoption adaptation processes and informing policy development that links AI integration with long-term workforce sustainability in medical imaging.

## Introduction

AI technologies are rapidly being integrated into medical imaging to enhance clinical efficiency, resilience, and precision [1]. This integration is reshaping medical imaging workflows and professional practices, including training [2]. Understanding medical imaging workforces’ beliefs, attitudes, intentions, and behaviors toward AI adoption is critical to ensure a meaningful integration of AI into the real-world clinical environment, including ensuring that both the financial and human resource (HR) costs and benefits of this major change are properly considered [3].

Workforce technology adoption is a mature but burgeoning research field, with multiple theoretical models used to explain the factors that influence users’ adoption and adaptation of new technology [4]. One key theory, for example, is the Technology Acceptance Model (TAM), which posits that perceived usefulness and perceived ease of use shape users’ attitudes and behavioral intentions toward a technology. Technology adoption or acceptance and adaptation behaviors are interrelated and associated with long-term outcomes, including improved productivity. Employees’ interactions with new technologies can shape their postadoptive adaptation behaviors, including how they modify system use and respond to technology-related changes over time [5-7]. Sun [6], for example, suggested that postadoptive behaviors involve a cycle of adaptation in which employees modify their use of new technologies over time. Similarly, Beaudry and Pinsonneault [7] argued that employees choose different adaptation strategies based on the expected outcomes of the technology event and the assessment of their control over the situation. Additionally, organizational sustainability of technology adaptation is commonly defined by how adopted new technologies improve business models to better address societal, environmental, and economic challenges [8,9]. This application of organizational sustainability has not yet been adequately considered in medical imaging; however, the rapid uptake of AI systems in radiology departments makes such considerations critical to the integrated workforce’s adoption. In this study, postadoption adaptation in radiology refers to the cognitive, behavioral, and workflow adjustment undertaken by clinicians following the initial acceptance and adoption of AI-driven systems.

AI offers unique advantages in aiding diagnostic pathways by reducing workloads [10,11], preventing overdiagnosis, and reducing reliance on radiologists [12]. This transformation is particularly welcome, given significant workforce shortages and increased demand for radiologists reported across many health services [13-15]. Research to date indicates that the technology innovation-behavioral theories employed in AI-driven diagnosis and treatment have examined the antecedents in isolation [16-18]. This offers a limited understanding of how AI acceptance decisions develop into postadoption processes that promote workforce sustainability.

More specifically, Perez et al [19] reported that little is known about how radiologists adapt to AI systems and how they cope with its associated threats, challenges, and opportunities. Malamateniou et al [20] also identified that perceptions of radiology AI system adoption and postadoption adaptation dynamics have not yet been sufficiently realized to ensure safe usage of this organizational change process. Unsurprisingly, Sesay et al [21] showed that IT implementation consequences emerge gradually through cascading phases shaped by organizational structures and policy constraints. Organizational pressures, including financial pressures, have been associated with changes or declines in employees’ adaptation behaviors [22]. These studies suggest that continuous adaptive efforts (eg, proactively seeking training in AI applications and conducting robust postimplementation auditing and clinical validation) are essential to unlocking the full value of new technologies, optimizing effectiveness, and supporting sustainable workforce outcomes.

Existing evidence describes, to varying extents, how AI adoption is linked to adaptation and workflow adjustments. However, it is not clear exactly how radiologists’ adaptation behaviors to an AI system are associated with skills, retention, task performance during AI-assisted practice, diagnostic accountability, and long-term professional resilience, thereby supporting radiologist workforce sustainability. This gap in understanding the adoption-to-sustainability pathway motivates the current review, which addresses the following research question:

1. What workforce factors shape AI adoption and postadoption adaptation among medical imaging workforces, and how are these factors linked to workforce sustainability outcomes?

## Methods

### Overview

To answer the research question, a systematic literature review was conducted. The systematic review followed the 5 steps of the PRISMA (Preferred Reporting Items for Systematic Reviews and Meta-Analyses) guidelines: identification of information sources; development of the search strategy; application of inclusion and exclusion criteria; screening and data extraction; and assessment of study quality and risk of bias [23], which are discussed in detail as follows.

### Information Sources

This systematic review searched for peer-reviewed journal articles within 7 digital databases: Embase, PsycINFO, Medline, CINAHL, Web of Science, ScienceDirect, and Scopus that described AI research in medical imaging published between January 2020 and September 2025.

### Search Strategy

Seven digital databases were selected based on their extensive coverage of AI in medical imaging research and their indexing of high-impact journals. Searches were conducted iteratively across databases between 25 August and 1 September 2025. A combination of keywords relating to AI and radiology was used across all databases, comprising “artificial intelligence,” “AI,” “machine learning,” “deep learning,” or “neural network*” combined with “radiology*” or “radiologist*.” These were further combined, in line with each database’s indexing structure and controlled vocabulary, with terms drawn from the following list: “adapt*,” “accept*,” “trust,” “role change,” “role transformation,” “professional identity,” “burnout,” “workload,” “job satisfaction,” “resist*,” “technology acceptance,” “implementation,” “integration,” “adoption,” “readiness,” “perception*,” “attitude*,” “decision support,” “workflow,” “satisfaction,” “experience*,” “impact,” “effect*,” “evaluation,” “preparedness,” “competence,” “clinical practice,” “barrier*,” “facilitator*,” “challenge*,” “confidence,” “anxiety,” “usability,” “fear,” “engagement,” “collaboration,” “autonomy,” “self-efficacy,” “leadership,” “morale,” and “organizational culture.” Population and workforce terminology varied according to the indexing and syntax of each database and, where supported, included terms such as “medical imaging professional,” “radiologic technologist,” “health personnel,” “allied health personnel,” “workforce,” “clinician*,” “physician*,” “medical professional*,” and “healthcare professional*,” in addition to “radiolog*” and “radiologist*.” These broader terms were used to capture professionals interacting with AI within medical imaging contexts, with population eligibility subsequently determined during screening. Database-specific filters, such as publication date range and document type (eg, research articles in Scopus), were applied where available. The full search strings for all 7 databases are presented in Table S1 in Multimedia Appendix 1.

### Inclusion and Exclusion Criteria

The inclusion and exclusion criteria for this review were designed to align with the study’s focus on AI adoption, adaptation behaviors, and workforce sustainability in medical imaging. Studies were included if they were peer-reviewed English-language journal articles published between January 2020 and September 2025 in journals ranked in Quartile 1 (Q1) or Quartile 2 (Q2) (Q1 or Q2) according to the SCImago Journal Rank, and addressed radiologists’ AI adoption and adaptation in medical imaging. The Q1 or Q2 criterion formed part of the predefined eligibility criteria and was applied during title and abstract screening. Journal eligibility was subsequently verified during full-text assessment before final inclusion. Eligible studies examined AI, machine learning, or deep learning applications across imaging modalities, including magnetic resonance imaging (MRI), computed tomography (CT), X-ray, ultrasound, positron emission tomography (PET), single-photon emission computed tomography (SPECT), mammography, and image-based endoscopy, in human subjects. More specifically, studies were eligible if medical imaging workforces interacted with or evaluated AI system outputs as part of their clinical or diagnostic role (eg, as readers, interpreters, or evaluators), regardless of whether the primary outcome measured was attitudinal, behavioral, or performance-based. Studies were excluded if AI models were developed or validated using only imaging data with no evidence of professional interaction with system outputs. Articles presenting empirical evidence using quantitative, qualitative, or mixed methods approaches were also included. In addition, research examining technological, clinical, organizational, operational, and behavioral dimensions of AI adoption and adaptation in medical imaging was included. In contrast, studies were excluded if they were review articles, conference papers, book chapters, books, editorials, opinions, research notes, industry reports, or government documents. Studies focused on AI adoption and adaptation outside the medical imaging field, those not involving human participants, and those using purely technical, conceptual, or simulation-based models were also excluded.

This review included and retained both workforce perception or attitude studies (eg, surveys, interviews assessing trust, readiness, and autonomy) and diagnostic performance studies involving radiologist participation (eg, reader studies comparing AI-assisted vs unassisted performance) as distinct but complementary evidence types. Performance studies were retained to capture immediate task-level outcomes arising from professional interaction with AI-assisted medical imaging systems, including workload, reading time, diagnostic accuracy, and sensitivity (Table 1, Performance subtheme). These immediate performance outcomes were treated as distinct from longer-term workforce adaptation and sustainability outcomes and were not interpreted as direct evidence of either construct. They are complementary to studies on workforce members’ self-reported perceptions and attitudes (Table 2). Accordingly, studies that included model development or validation were retained only when they also examined professionals’ interaction with AI outputs, such as reader studies in which imaging professionals interpreted cases with AI assistance or could reconsider their initial decisions using AI-generated predictions, scores, or visual guidance. Purely technical model-performance evidence was not sufficient for inclusion. Both evidence types were therefore included as necessary components of the workforce sustainability picture.

### Screening, Data Extraction, and Synthesis

Records identified across the 7 databases were imported into Covidence, where 21,685 duplicate records were automatically identified and removed prior to title and abstract screening. Three reviewers (MKZ, NH, and JP) screened the titles and abstracts, followed by a full-text review of the selected studies. The authors resolved any variations in study selection and inclusion via discussions, and formal interrater agreement statistics (eg, Cohen kappa) were not calculated. One member of the author team extracted data from all 69 included studies using a structured form with predefined fields. These covered study identification and bibliographic details, country, study design, sample characteristics, radiology service or clinical focus, imaging modality, AI methods and systems, theories used or mentioned, workforce attitudes toward AI implementation and use, HR, and workforce sustainability outcomes. A second member (KMZ) independently conducted a confirmatory review of the extracted data. Any discrepancies were discussed and resolved by consensus. The final extracted data were then used for descriptive analysis and thematic coding. This review used multiple software packages to strengthen methodological rigor. This included Covidence for the initial literature search, screening, and data extraction, SPSS to produce descriptive statistics, and NVivo (Lumivero) for thematic analysis.

Given the methodological diversity of included studies (quantitative, qualitative, and mixed methods) and the review’s aim of synthesizing perceptions, behaviors, and conceptual relationships, this review adopted a systematic review design with thematic synthesis, consistent with Thomas and Harden [24]. This approach produces an interpretive synthesis rather than scoping and a descriptive map of the literature. Thematic coding followed an inductive approach and was derived from the data (extracted study findings); no a priori codebook was developed, consistent with this inductive design. Coding was conducted by a single reviewer (JP) using NVivo, based on data-extraction fields that inductively captured key findings, workforce attitudes, and HR and workforce outcomes for each of the 69 included studies. Initial codes were generated from extracted text, then iteratively grouped into subthemes and higher-order themes. For example, a study reported that radiographers valued AI’s diagnostic support while also expressing concern about deskilling was coded under both “Perceived benefits” and “Perceived threat” to professional role and identity’ (Table 2); a study reporting reduced reading times alongside unmet training needs was coded under both “Performance” and “Training and skills development” (Table 1). Emerging themes and their description were periodically reviewed and discussed with the wider author team to check interpretive consistency, though formal interrater coding reliability was not calculated as coding was conducted by a single reviewer. To strengthen verification, a second member of the review team (KMZ) independently conducted a confirmatory review of the study-to-theme coding. The reviewer agreed with the overall thematic structure, with minor differences relating primarily to theme labeling rather than substantive coding. These differences were discussed and resolved by consensus and did not alter the final thematic structure or study-to-theme assignments. Study frequency (n) for each subtheme reflects the number of studies in which that theme was substantively coded; as studies could be coded under multiple subthemes where their findings addressed multiple constructs, subtheme frequencies are not mutually exclusive and do not sum to 69. These frequencies represent counts of thematic occurrences across the literature and should not be interpreted as effect sizes or as the strength of association. A higher frequency indicates a theme was more commonly discussed across the included studies.

In this review, workforce sustainability is used as an overarching concept referring to the capacity of the radiology workforce to maintain effective, healthy, and adaptive functioning in response to AI integration over time. As this review used an inductive thematic synthesis, the specific dimensions constituting workforce sustainability were not predetermined but identified inductively from the included literature (as presented in the Results section), and synthesized into a conceptual framework (Figure 1). Accordingly, job satisfaction and work engagement are presented as the workforce-sustainability-related outcomes that emerged from the reviewed evidence, rather than as an exhaustive or validated set of dimensions of workforce sustainability.

**Figure 1.**
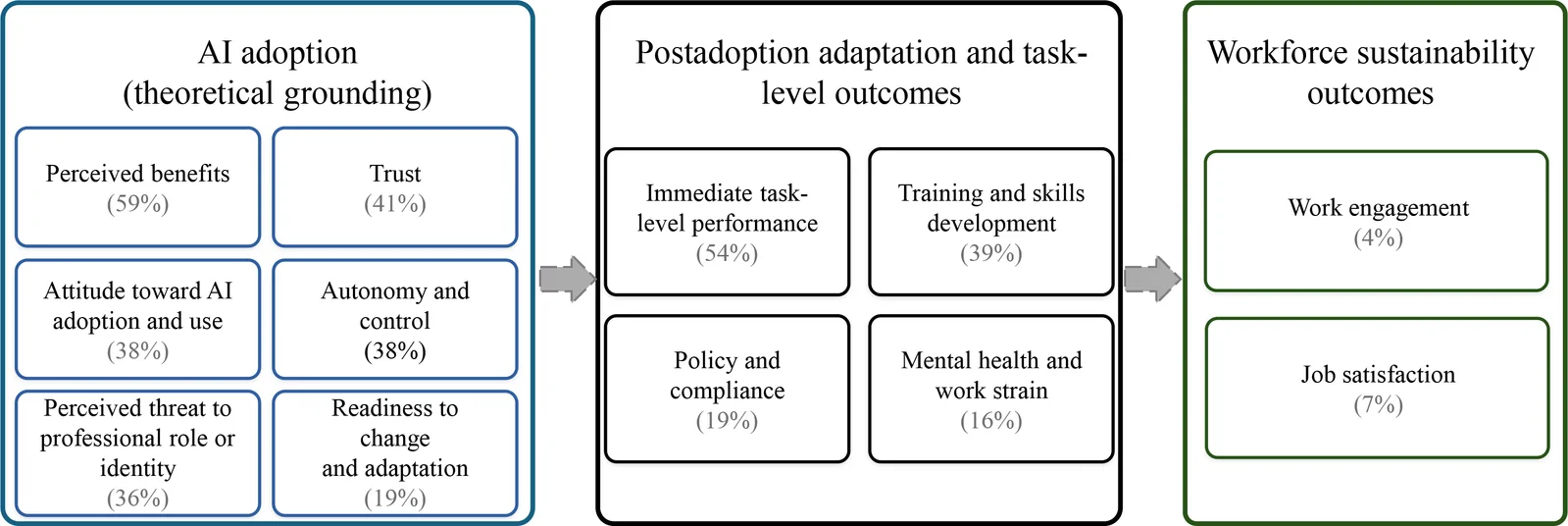
AI adoption-adaptation for workforce sustainability in medical imaging. Percentages indicate the proportion of included studies (N=69) in which each theme was coded. Frequencies do not represent effect sizes. Immediate task-level performance refers to short-term diagnostic and workflow outcomes during AI-assisted practice and is distinct from longer-term workforce adaptation and sustainability outcomes. This framework is presented in proposition format; relationships depicted have not been empirically tested and should not be interpreted as causal.

### Quality Assessment

This study conducted a quality assessment using the mixed methods appraisal tool (MMAT) to cover qualitative, quantitative, and mixed method articles [25]. MMAT evaluates the methodological quality of the literature in terms of appropriateness, sampling techniques, completeness, and reporting of study outcomes [26]. The initial MMAT appraisal was conducted by one member of the review team (JP) and subsequently checked by another member (KMZ). Minor differences in item-level judgments were discussed between the 2 reviewers and resolved by consensus through review meetings. Consistent with the MMAT 2018 guidance, we did not calculate an overall score or assign an aggregate quality category from the individual criteria. Instead, each study was assessed using the 2 screening questions and the 5 design-specific MMAT criteria, with judgments reported as “Yes,” “No,” or “Can’t tell” in Tables S3-S5 in Multimedia Appendix 1. These criterion-level judgments were not used to exclude studies or guide thematic coding. Rather, they informed the interpretation of the methodological strengths and limitations of the included evidence, as discussed in the Contributions and Limitations section. Detailed item-level MMAT assessments for each included study, based on criteria such as clarity of research questions, appropriateness of collected data, sampling strategy, representativeness, measurement validity, risk of bias, and statistical analysis, are presented in Tables S3-S5 in Multimedia Appendix 1.

## Results

### Overview

This review presents the frequency of the records, in accordance with PRISMA guidelines (Figure 2), for each stage of the study classification process. The review yielded a final sample of 69 articles published across 37 journals that met the study’s inclusion criteria. The quartile 1 (Q1) or quartile 2 (Q2) journal criterion was applied during title and abstract screening and checked again at full-text assessment as an additional eligibility check. The 4 studies excluded based on journal-quartile eligibility shown in Figure 2 represent only those identified at full-text assessment, rather than the total number excluded based on this criterion throughout the screening process.

**Figure 2.**
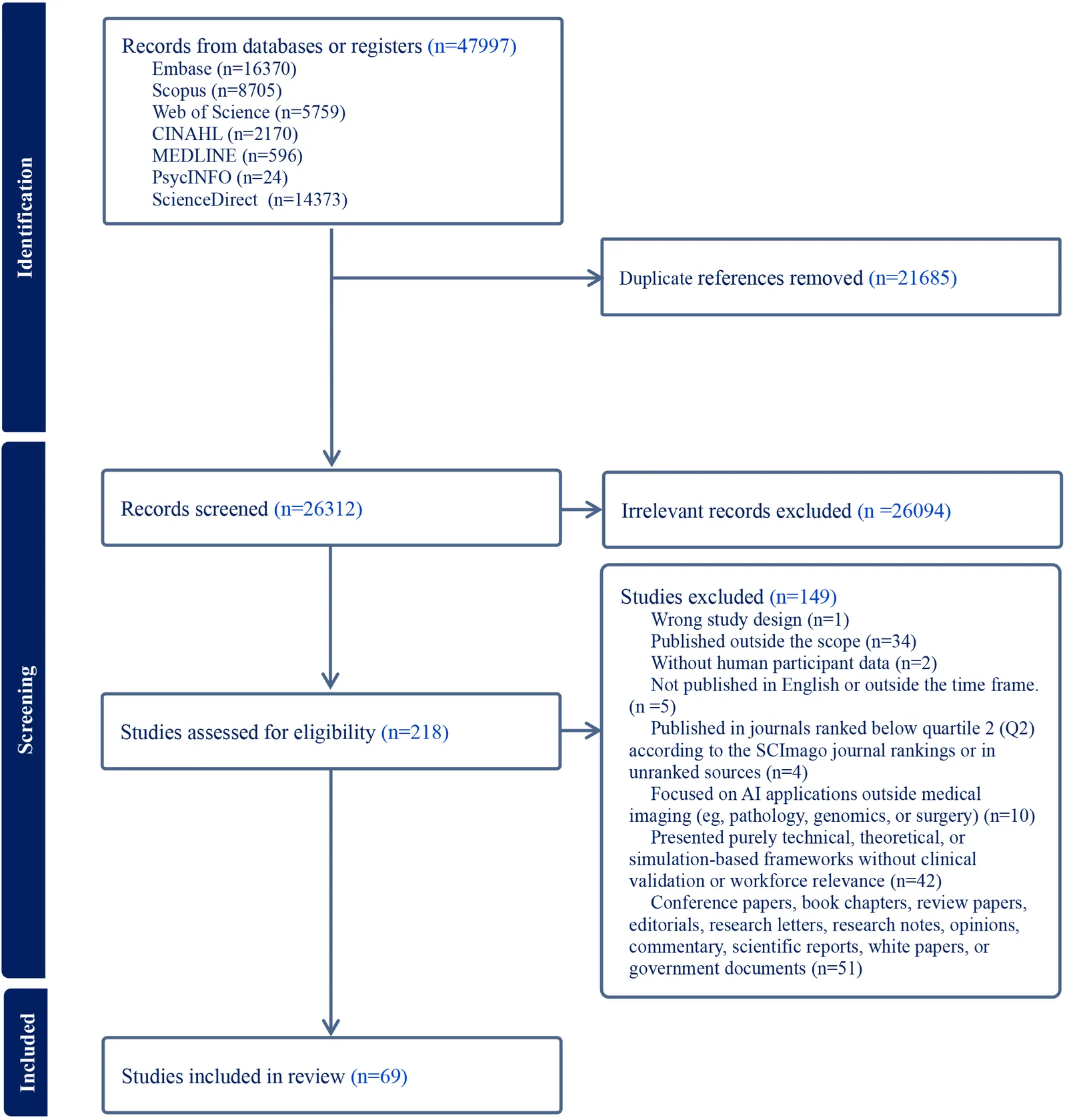
Literature search and selection process using the PRISMA (Preferred Reporting Items for Systematic Reviews and Meta-Analyses) guidelines.

### AI Adoption and Adaptations in Medical Imaging

Table 1 summarizes the literature on workforce perceptions and attitudes toward AI adoption and postadoption behaviors in clinical imaging. Forty-one articles identified perceived benefits as a key antecedent of AI radiology adoption (41/69, 59%) [3,14,15,18,19,27-62]. A significant proportion of the studies (28/69, 41%) [11,18,19,28,31,32,34-36,38,42,48-61,63-65] centered on “trust” in the integration of AI into clinical practice.

Twenty-six empirical studies (26/69, 38%) [3,8,11,15,18,19,32,35,41-47,49,50,52,55-59,61,66,67] assessed the workforce’s explicit perceptions, enthusiasm, optimism, and willingness to adopt AI in clinical practice, focusing on how AI can support them, for example, by reducing scan times, improving diagnostic accuracy, and easing their workload. Collectively, the authors of these studies emphasized that the medical imaging workforce enthusiastically adopted AI-embedded systems, with curiosity and acknowledgment of their potential to support their work. A similar number of articles (26/69, 38%) [3,11,18,19,27,28,31-33,36,41,47-50,52,54-56,58,59,61,62,64,65,67] reported that radiologists’ perceptions of autonomy (ie, job control) were associated with AI-assisted clinical outcomes, especially regarding the desire to retain authority over diagnostic decisions and interpretations of AI outputs. Twenty-five articles (25/69, 36%) [3,8,18,19,28,34,35,41,43-52,55,56,58,59,61,66,67] described workforce concerns that AI implementations could negatively affect their professional roles, expertise, and identity. These concerns centered on radiologists’ deskilling, loss of autonomy, erosion of professional reputation, and uncertainty about future career prospects. Finally, 13 studies (13/69, 19%) [18,19,35,42,43,45,48,49,51,52,55,58,65] discussed adaptation and readiness to change, willingness to engage with technological advancements, and integration of AI into clinical practice. Overall, the authors highlighted several factors that were associated with AI adaptations in medical imaging, including a positive attitude toward learning and acquiring new knowledge and skills, recognition of ongoing role changes, perceived readiness, and the role of organizational and peer support for the AI system.

**Table 1.** Thematic analysis of workforce perceptions and attitudes toward AI adoption and adaptations in medical imaging.

Workforce perception and attitudes category	Thematic description	Study frequency (n)	Study coverage (%)^a^	Representative literature
Perceived benefits	The subtheme focuses on the perceived advantages and value of AI in radiology and related imaging practices. It encompasses improvements in diagnostic accuracy, workflow efficiency, workload reduction, clinical decision support, and the overall enhancement of professional performance.	41	59	[3,14,15,18,19,27-62]
Trust	Trust reflected in the included studies as the degree of confidence stakeholders (eg, radiologists, radiographers, and clinicians) place in AI systems’ diagnostic and decision-making outputs. It is reported quantitatively (eg, percentages expressing trust, distrust, or neutrality) and qualitatively (personal statements of confidence or doubt). The studies link trust to factors such as perceived accuracy, transparency, evidence-based validation, explainability, and the risk of overreliance or automation bias. Trust (or the lack thereof) is described as directly influencing users’ intention to adopt, rely on, or reject AI in clinical practice.	28	41	[11,18,19,28,31,32,34-36,38,42,48-61,63-65]
Attitude and intention toward AI adoption and use	The subtheme captures health care workforces’ explicit perceptions, enthusiasm, optimism, and willingness to implement AI in clinical practice. Across the reviewed studies, participants generally expressed positive attitudes toward AI and intentions to use it, including excitement, curiosity, and recognition of its potential to support or complement their work.	26	38	[3,8,11,15,18,19,32,35,41-47,49,50,52,55-59,61,66,67]
Autonomy and control	Autonomy and control refer to the degree to which health care workforces, particularly radiologists and radiographers, retain authority over diagnostic decisions and the interpretation of AI outputs. This includes maintaining oversight of AI recommendations, the ability to modify or reject AI-generated results, and ensuring accountability for patient care outcomes. The subtheme also covers perceptions of AI as an adjunct rather than a replacement with professional decision-making autonomy and responsibility central to safeguarding accuracy, patient safety, and legal or ethical standards.	26	38	[3,11,18,19,27,28,31-33,36,41,47-50,52,54-56,58,59,61,62,64,65,67]
Perceived threat to professional role and identity	The subtheme reflects health care workforces’ concerns that AI implementation may negatively impact aspects of their professional roles, expertise, and identity. It includes fears of job displacement, reduced demand, deskilling, loss of autonomy, and erosion of professional reputation, as well as uncertainty about future career prospects. These threats are expressed across both radiologists and radiographers with variation according to experience, AI knowledge, and training.	25	36	[3,8,18,19,28,34,35,41,43-52,55,56,58,59,61,66,67]
Readiness to change and adaptation	The subtheme refers to health care workforces’ explicit acknowledgment of evolving professional roles, willingness to engage with technological change, and to incorporate AI into practice. This includes positive attitudes toward learning new skills, recognition of ongoing role evolution, perceived preparedness, and the influence of organizational or peer support on adapting to new technologies. The subtheme also captures reported barriers such as resistance, withdrawal, or limited confidence.	13	19	[18,19,35,42,43,45,48,49,51,52,55,58,65]

a The percentages are calculated based on the included articles (N=69). Approximately 30% of the studies (n=21) did not investigate this theme and instead focused on human resource and workforce sustainability.

### Workforce Sustainability in AI Adoption and Adaptation

Moving beyond AI as a mere diagnostic tool, it is also important to evaluate how this advanced technology supports the development of a sustainable medical imaging workforce. Evidence from the descriptive characteristics and thematic analysis (Table 2) identified 37 studies (37/69, 54%) where AI-assisted systems were associated with shorter radiologic reporting and reading times, while also being associated with higher detection of true-positive cases and accuracy of lesion identification [14,18,27-39,63,64,68-87]. For example, the professional-interaction evidence in reader studies [68,69,84] involved radiologists interpreting imaging cases with AI assistance and using AI-generated predictions, scores, or visual guidance to inform or reconsider their diagnostic decisions.

An additional number of 27 studies (27/69, 39%) corroborated the need for educational activities, programs, and resources to help medical imaging workforces adapt to and acquire new knowledge and skills for the use of AI [3,8,11,15,18,19,33,40-56,65-67]. The authors identified the significant lack of curricula, mentorship, and access to courses as direct impediments to a sustainable workplace. Thirteen studies (13/69, 19%) [15,19,33,34,36,46,48-52,54,57] also recognized that sustainable workforce development within clinical workflows relies on supportive organizational policies and guidelines, as well as regulatory compliance practices. These studies highlighted examples of unclear regulatory standards and ethical guidelines, which raised skepticism and created resistance among medical imaging workforces concerning the use of AI-assisted systems. A total of 11 articles (11/69, 16%) [11,15,19,33,34,40,45,48,50,57,70] examined mental health and work strain among medical imaging workforces, including reduced psychological well-being, and reported experiences of stress, burnout, anxiety, fatigue, and cognitive strain. In contrast, several articles reported that AI-embedded systems were associated with lower perceived mental strain and radiologists’ anxiety, alongside support for informed decision-making and lower repetitive task burdens; although these impacts were reduced in situations of high workloads and low AI acceptance [11,15,19,33,34,40,45,48,50,57,70].

Furthermore, 5 studies (5/69, 7%) [11,18,19,33,47] reported medical imaging workforces’ optimism toward AI integration, that is, improved diagnostic efficiency, more informed interactions with patients, and access to adequate technical support resources. However, these authors also note that employees’ levels of job satisfaction could decline when medical imaging workforces have limited knowledge or negative attitudes toward AI adoption. Similarly, 3 articles (3/69, 4%) [19,28,57] highlighted the significance of medical imaging workforces’ perceptions of work engagement and acknowledged the perceived functional, social, emotional, and cognitive value of AI use, along with opportunities for learning and collaboration. In addition, their active engagement with AI-assisted systems and related tasks, including attention to system use and proactive participation in AI-related training, was reported alongside greater adaptation to AI-assisted system changes, and was discussed in relation to workforce sustainability [19,28,57].

The limited number of studies addressing these outcomes indicates that the workforce-sustainability evidence base remains underdeveloped. These themes therefore represent the sustainability-related outcomes identified in the available literature rather than a comprehensive specification of workforce sustainability.

**Table 2.** Thematic analysis of immediate task-level performance, adaptation-related factors, and workforce sustainability outcomes in relation to AI adoption.

Outcome or adaptation-related category	Thematic description	Study frequency (n)	Study coverage (%)	Representative literature
Immediate task-level performance	Performance refers to measurable improvements in diagnostic accuracy, sensitivity, specificity, efficiency, and workflow optimization among radiologists when assisted by AI systems or algorithms. This includes reductions in interpretation, reporting, and reading times; increased detection of true-positive cases; enhanced diagnostic confidence; and improvements in overall accuracy and lesion identification. Performance outcomes vary by radiologist experience, AI model type, and clinical context but consistently demonstrate time savings, accuracy gains, or both when AI tools are appropriately integrated. These outcomes reflect short-term diagnostic and workflow performance during AI-assisted practice and are not interpreted as direct evidence of longer-term workforce adaptation or sustainability.	37	54	[14,18,27-39,63,64,68-87]
Training and skills development	The subtheme refers to the educational activities, programs, and resources that enable medical imaging practitioners to acquire, maintain, and enhance knowledge and competencies related to AI in medical imaging. This includes foundational AI principles, practical application of AI tools, ethical considerations, programming skills, and continuous professional development across career stages. The subtheme also reflects on barriers to learning, such as a lack of formal curricula, mentorship, access to courses, or time, as well as motivations for engaging in AI training to maintain professional competence.	27	39	[3,8,11,15,18,19,33,40-56,65-67]
Policy and compliance	Policy and compliance refer to regulations, guidelines, and organizational measures that govern AI use in radiology. The results show that physicians and radiologists expect AI to be regulated by specific policies and certified for clinical use, and that clear regulations are necessary to reduce uncertainties about ethical and legal responsibility for clinical outcomes. When such regulatory standards are absent or unclear, health workforces in radiology may avoid using AI-assisted tools.	13	19	[15,19,33,34,36,46,48-52,54,57]
Mental health and work strain	The subtheme captures how the integration and use of AI tools in radiology affect employees’ psychological well-being, including stress, burnout, anxiety, fatigue, and cognitive strain. Evidence indicates that AI can both mitigate mental strain by reducing anxiety, supporting decision-making, and alleviating repetitive task burdens, and also exacerbate it, particularly when high workload and low AI acceptance are present. The subtheme also reflects the role of AI in influencing confidence, performance consistency, and coping with demanding tasks.	11	16	[11,15,19,33,34,40,45,48,50,57,70]
Job satisfaction	Job satisfaction in the included studies is reflected in radiologists’ work enjoyment and contentment with both workflow and organizational support. Satisfaction is influenced by factors such as optimism toward AI integration, enhancement of diagnostic efficiency, interaction with patients, and access to adequate technical resources. Job satisfaction may decrease when radiologists have limited knowledge or negative attitudes toward AI implementation.	5	7	[11,18,19,33,47]
Work engagement	The subtheme in the included studies is reflected as users’ and clinicians’ engagement with AI systems and related tasks, including attention to system use and proactive participation in AI-related activities. The included studies indicate that engagement is supported by features that encourage critical evaluation (eg, visibility of system confidence levels); perceived functional, social, emotional, and cognitive value of AI use; and opportunities for learning and collaboration. Engagement is further demonstrated through behaviors such as seeking information, participating in AI projects, and joining working groups, which can help users remain involved despite apprehension.	3	4	[19,28,57]

### Descriptive Characteristics of the Reviewed Studies

The descriptive characteristics of the reviewed studies are summarized in Table S2 in Multimedia Appendix 1. The temporal distribution of the reviewed studies indicates an increasing academic interest in adopting AI within medical imaging, with published studies ranging from just 2 in 2020 (2.90%) [47,61] to 21 articles published in 2024 (30.43%) and 18 articles published in 2025 (26.09%).

The reviewed studies were concentrated in Asia, the United States, and Europe. Notably, a large number of studies originated from China (n=14, 20.28%), followed by multinational studies (n=10, 14.49%), the United States (n=7, 10%), and Germany (n=7, 10.14%). Fewer studies originated from the United Kingdom, Europe, South Korea, and Saudi Arabia, and several articles were published in multiple countries.

It is also apparent that the reviewed studies primarily used quantitative research designs (n=51, 73.91%), followed by a small number of mixed-method studies (n=12, 17.39%) and only 6 qualitative studies (8.70%). The reviewed studies included diverse participant groups, including radiologists, radiographers, medical students, and mixed groups. General radiology accounted for the largest percentage of these reviewed studies (n=16, 23.18%), followed by breast imaging (n=13, 18.84%), neuroradiology (n=10, 14.49%), and chest and thoracic radiology (n=8, 11.59%). Most of these articles assessed AI systems based on MRI (n=11, 15.94%), CT (n=10, 14.49%), and X-rays (n=9, 13.04%), or on a combination of these modalities.

The organizational and information systems theories used to examine medical imaging workforce attitudes and behaviors toward AI adoption, adaptation, and organization are classified in Table S2 in Multimedia Appendix 1. However, only 10% of the reviewed studies (n=7) explicitly applied a theoretical framework. The cited theories included the TAM [88], Theory of Diffusion of Innovation [89], Nonadoption, Abandonment, Scale-up, Spread, and Sustainability (NASSS) framework [90], the model of workflow integration based on the SEIPS (Systems Engineering Initiative for Patient Safety) model [91], Consumption values theory [92], Involvement theory [93], Identity Work theory [94], and Job Crafting theory [95]. The majority of included articles (n=59; 85.50%) did not empirically apply any theoretical framework in their research design or analysis. A total of 69.57% (n=48) of the reviewed studies explicitly investigated workforces’ attitudes and behaviors toward the AI adoption they were experiencing, although nearly all the reviewed studies claimed to be assessing workforce sustainability (n=66, 95.65%).

## Discussion

### Principal Findings

The findings of this review highlight that published research on AI implementation in radiology departments has predominantly focused on perceptions of AI adoption, rather than the consequences of AI adaptation and workforce sustainability outcomes. This reveals an obvious research imbalance, with a current predominant focus on AI integration into workflows as an initial acceptance decision rather than on the longer-term evaluation of the organizational adjustment process and patient outcomes. Specifically, organizational postadoption adaptation processes, including AI radiology workflow integrations, radiologists’ AI coping strategies, professional training, and skill development, are significantly underexamined. Furthermore, limited empirical evidence links the theoretical constructs of medical imaging AI adoption and adaptation with workforce sustainability outcomes, a significant current research oversight.

The findings of this review were synthesized into a conceptual model of AI adoption-adaptation for workforce sustainability in medical imaging (Figure 1). The 3 main components of this model are discussed below and are suggested as the key components for future research to examine to produce improved evidence relating to the sustainability of the medical imaging workforce during the introduction of AI systems. Throughout this discussion, findings reported by included studies are presented with supporting citations, while broader interpretations and propositions offered by the review authors, extending beyond what individual studies report, are flagged using interpretive language (eg, “we argue,” “we posit,” “this suggests”).

This framework is presented as a hypothesis-generating conceptual model derived from thematic synthesis, rather than a tested or validated causal pathway. The relationships depicted have not been empirically tested within or across the included studies and should be treated as propositions for future research rather than confirmed sequences. More specifically, the 3 groups shown in Figure 1 are conceptually linked through an interdependent process as denoted by the double-headed arrows. We categorized the themes as AI Adoption factors, such as technological perceptions (eg, perceived benefits), cognitive evaluations (eg, trust and attitudes), and professional considerations (eg, autonomy, identity, and readiness to change), which were discussed in relation to initial engagement with AI systems. These adoption-related themes were conceptually considered alongside postadoption adaptation factors, including training and skill development, work strain, and policy and compliance, as well as immediate task-level performance during AI-assisted practice. Immediate task-level performance was treated as a distinct short-term outcome and not as direct evidence of longer-term workforce adaptation or sustainability. In turn, these adaptation processes were conceptually considered alongside workforce sustainability-related outcomes identified in the reviewed literature, namely work engagement and job satisfaction. These conceptual relationships should not be interpreted as empirically established directional or causal effects. Rather, the groupings reflect an interpretive synthesis of themes identified across the literature and are not relationships inferred from thematic frequencies. Although the included studies have examined the factors of these 3 dimensions, their interrelationships across adoption, adaptation, and sustainability have not been empirically tested, highlighting a critical knowledge gap in the current literature. Thus, we conceptualize their interrelationships as part of a unified process of AI adoption, adaptation, and sustainability in real-world clinical imaging environments for future research.

### AI Adoption in Medical Imaging

We identified 6 key factors related to AI adoption in medical imaging: perceived benefits, trust, readiness to change, autonomy, perceived threat to professional identity, and attitudes toward AI adoption and use. Issues concerning medical imaging workforces’ degree of trust, professional identity threat, and autonomy were assessed in 41% (n=28), 36% (n=25), and 38% (n=26) of the 69 reviewed articles, respectively, indicating that these concerns are recurrent themes in the literature. This is balanced by the research respondents in these reviewed studies who did express excitement and curiosity about medical imaging AI introduction. Across the reviewed studies, positive attitudes toward AI’s potential were reported alongside concerns about its implementation, suggesting a pattern of cautious optimism within the available evidence [8]. Importantly, several reviewed studies reported limited fear of job displacement, while concerns were more often framed around nontechnical aspects of AI adoption. The evidence from the findings reinforces the position that social and professional factors may shape AI adoption alongside perceptions of its technical value. The limited discussions and lack of evolution of organizational policies concerning AI usage, ethical concerns, and job fears serve to hinder the adoption of this technology. Thus, the reviewed studies reported favorable perceptions of AI adoption alongside persistent social, professional, organizational, and governance concerns that may shape adoption.

Surprisingly, a large portion of the reviewed studies are not explicitly informed by any theoretical explanation. This, we suggest, is imperative to produce evidence-based conclusions about medical imaging workforces’ acceptance, willingness, and trust in AI systems. Given the complex and advanced technology offered by AI systems in medical imaging, it is essential that medical imaging workforces’ acceptance or rejection behaviors should be thoroughly examined within validated theoretical frameworks. For instance, future research can be informed by the integration of IT Adaptation theory [5] and the coping model of user adaptation (CMUA) [7] to examine AI adoption-adaptation for workforce sustainability. Researchers also strongly recommend future studies to empirically validate theories of AI acceptance and trust in medical imaging. This validation will advance our understanding of how AI adoption translates into adaptation behaviors and long-term workforce sustainability.

### Postadoption Adaptation of AI in Medical Imaging

The evidence emerging from this literature review also identified 4 adaptation patterns important for long-term workforce sustainability: training and skills development, task performance during AI-assisted practice, mental health and occupational stress, and policy and compliance. Although AI-assisted systems were examined across multiple studies, radiologists’ cognitive, behavioral, and workflow adjustments to these technologies were less frequently assessed, a reflection of minimal discourse in the medical imaging space. The disproportionate lack of attention to “readiness to change and adaptation” within the existing literature suggests a weighting of technical aspects over the humane. We argue that adaptations to a new technology require more than initial acceptance but also require sustained learning and a reinterpretation of professional identity.

For example, Bhattacherjee and Harris [5] discussed how successful technology adaptations can be measured using 3 antecedents of adaptation: usefulness, ease of adaptation, and IT adaptability. This theoretical lens can be translated into the context of AI and medical imaging. Successful adaptations involve employees’ knowledge and skill development, performance improvements, assessments of mental health and work strain, and favorable policy implementation. Additionally, it is apparent that the workforce with AI training typically perceived AI systems as personally useful work tools, compared to employees with no AI training, who had a greater fear of AI replacement and thus lower acceptance of this adoption [44].

Knowledge and skill development emerged as an important theme in the context of AI adaptation in medical imaging. The reviewed literature indicated that some medical imaging workforces feared that AI implementation would lead to their job displacement, deskilling, and loss of professional identity [51]. More specifically, any fear of incompetence was overshadowed by radiologists’ fears of personal liability and AI arbitrations [67]. However, some studies showed that an AI-integrated system achieved improved results (specifically higher sensitivity) compared to a combined human-and-AI-based reading [72]. This review explains the lack of direct connection between adoption and adaptation behaviors, despite optimism about AI complementation. We posit that a slower rate of adaptation to AI in medical imaging workflows may be associated not only with job insecurity but also with fears about ethical standards and authority over AI outcomes.

Finally, “readiness to change” is also rarely assessed within the current literature, being included in only 19% (n=13) of the reviewed studies. Thus, while the reviewed studies reported both AI availability and trust-related findings, evidence concerning the organizational conditions needed to support longer-term adaptation remains limited. For example, the reviewed studies identified common barriers to AI-systems learning as being a lack of mentorship, time, funding, AI ethics education, privacy safeguards, and transparent algorithms [41,42]. These barriers may become more salient as the technical landscape of medical AI becomes increasingly heterogeneous and complex. Recent technical developments include multicenter learning approaches addressing data heterogeneity, generalization, and model interpretability across clinical settings [96], multimodal systems integrating clinical images, dermoscopic images, and textual information [97], and time-frequency and multiscale modeling for electrical impedance tomography (EIT)-based pulmonary disease diagnosis [98]. Although these technically oriented studies do not examine workforce adaptation or sustainability, they illustrate the increasing diversity and technical complexity of AI-enabled clinical systems and provide complementary context for considering how workforce training, interpretability, and adaptation may need to be examined across different clinical environments. This context is consistent with workforce concerns identified in the reviewed literature, including the ability to override AI decisions, explain opaque model outputs, and maintain their responsibility for patient outcomes. These systemic gaps in AI governance and clinical protocols create an epistemic environment unsuitable for meaningful organizational change processes.

### Workforce Sustainability in Medical Imaging

A key aim of this review was to assess how the adoption of AI medical imaging systems can support workforce sustainability. It is apparent that organizational processes which encourage critical evaluations of AI systems (eg, visibility of system confidence levels), perceived functional, social, emotional, and cognitive value of AI use, and opportunities for learning and collaboration were all discussed in relation to a sustainable clinical workforce [19,28,57]. Unsustainable legal and ethical standards, lack of training and educational infrastructure are associated with concerns regarding the vulnerability of the medical imaging workforce. Furthermore, the reviewed studies provided limited discussion of employees’ mental health, work engagement, job satisfaction, and skill development, which are each also associated with successful technology adaptation and its long-term sustainability.

The review results also revealed that few studies examined psychological burnout and deskilling anxiety [11,15,19,33,34,40,45,48,50,57,70], which can be correlated with lower acceptance and poor adaptation. These factors were identified as potential barriers relevant to adaptation and workforce sustainability. Such concepts (eg, deskilling) and their relationships with adaptation and workforce sustainability need further investigation. Deskilling anxiety and technostress may emerge as medical imaging departments plan to implement AI, particularly when professionals are concerned that its use could reduce the value of their existing skills, contribute to skill erosion, or affect their future career prospects. Examining these concerns may offer insight into their implications for workforce sustainability. Further research, particularly through in-depth qualitative or ethnographic approaches, could explore how medical imaging professionals experience, interpret, and respond to deskilling anxiety, technostress, and skill erosion as AI is introduced into practice.

We recommend that successful AI adaptation and the sustainability of an AI-equipped workforce require structured training, mentorship, and institutional support. Clear organizational AI policies and procedures can support the acceptance of AI-enabled medical imaging systems as part of routine radiographic practice and encourage their responsible use.

### Contributions and Limitations

This review assessed published empirical evidence concerning employees’ attitudes and perceptions of AI adoption, adaptation behaviors, and overall workforce sustainability, and offers 3 key considerations for future research. First, the findings offer a richer understanding of radiologic workforce perceptions and attitudes toward AI adoption and its conceptual links with adaptation behaviors and radiologic workflow. The findings provide valuable insights for health informatics, digital innovation, and behavioral researchers to understand the sustainable impact of AI systems on the medical imaging workforce. Second, this research found that the theoretical basis was not reported in the majority of published studies, a gap that requires immediate scholarly attention in future research.

Finally, this research proposed a unified conceptual framework (Figure 1) that offers an inclusive guideline and explanatory structure for understanding AI adoption and adaptation for medical imaging workforce sustainability. Without clear organizational policies and procedures, AI will remain an external imposition rather than an accepted facet of radiologic practice. This article provides a solid foundation for future research on radiologic workforce acceptance, easier AI adaptation strategies, and the sustainability of an AI-equipped workforce. Additionally, this review provides actionable insights for clinicians, stakeholders, and policymakers by identifying adaptation mechanisms that inform organizational strategies and policy development.

These findings have been interpreted with some limitations that suggest directions for future investigation. The included studies were also heterogeneous in design, population, and geographic setting, which limits direct comparability across findings. Relatedly, the criterion-level MMAT appraisal showed variation across specific methodological domains in the included studies. Few concerns were identified at the criterion level among the qualitative studies. In the quantitative studies, concerns centered mainly on sample representativeness and nonresponse bias. For mixed methods studies, variation was more apparent in the rationale for using a mixed methods design and in whether the individual components met the quality criteria associated with their respective methodological traditions. These criterion-level findings were used to contextualize the methodological strengths and limitations of the evidence base and were not used to weight or reclassify the thematic findings. No studies were excluded on the basis of the MMAT appraisal. Additionally, the thematic synthesis involved some researcher interpretation, with the initial coding conducted by one member of the review team (JP) before subsequent confirmatory review of the study-to-theme coding by another team member (KMZ). Meta-analysis was not conducted due to substantial heterogeneity in study designs, data types, and outcomes. An important direction for future research is to empirically test the proposed conceptual model (Figure 1) using quantitative methods (eg, structural equation modeling), to validate the hypothesized pathways between AI adoption, adaptation, and workforce sustainability.

A related methodological constraint concerns the scope of the literature searched. First, the review was restricted to English-language, peer-reviewed publications in Q1 and Q2 journals [99-101]. The journal-quartile criterion was established as an eligibility boundary and applied from the title and abstract screening stage, rather than being introduced at full-text assessment. The criterion defined the journal scope of the review and was not intended as a proxy for individual study quality. We acknowledge that journal quartile reflects journal-level citation standing rather than the methodological quality of individual studies; accordingly, study quality was assessed separately using the MMAT. The Q1 or Q2 restriction may nevertheless have excluded relevant evidence from lower-quartile or unranked journals and therefore represents a limitation on the comprehensiveness of this review. Consistent with Castañer and Oliveira [102], we frame this restriction as a boundary condition on our findings, which provides a conservative evidence base and may exclude relevant studies published in lower-quartile or nonindexed journals, potentially limiting the comprehensiveness of the review. We therefore recommend that future reviews without this constraint be conducted to verify whether excluded lower-quartile studies alter the thematic patterns identified here. This review was also not registered on PROSPERO (International Prospective Register of Systematic Reviews) or any other systematic review registry prior to conduct; we acknowledge this as a limitation of methodological transparency, and note that future reviews in this area would benefit from prospective registration. Second, the research also adhered to the selected timeframe of January 2020-September 2025, which may overlook data from earlier years. Third, this research only assessed AI adoption and adaptation and their impact on a sustainable AI-equipped medical imaging workforce. Future studies could also assess how the current evidence can be generalized to other AI-assisted systems and practitioners. Additionally, as this review’s thematic synthesis was inductive, workforce sustainability dimensions were derived from the included literature rather than being predetermined. Given that job satisfaction and work engagement were represented by relatively few studies, this component of the framework should be interpreted as a preliminary representation of the sustainability-related evidence available in the reviewed literature, rather than a comprehensive conceptualization of workforce sustainability. This limited evidence base is also reflected in the absence of distinct outcomes relating to retention and professional resilience. A promising avenue for future research is to theorize how AI-enabled work redesign shapes retention and professional resilience, including the organizational mechanisms and boundary conditions underpinning workforce sustainability.

### Conclusion

Despite an emerging trend toward widespread AI implementation in medical imaging, the research field remains relatively immature. The interrelationships among AI adoption, postadoption adaptation, and workforce sustainability are increasingly apparent yet remain poorly understood, as evidenced by the 6 adoption factors and 4 adaptation themes identified in this review, which have not yet been empirically tested as an integrated pathway. These findings suggest a need for health services to consider not only the adoption of AI medical imaging systems, but also the organizational conditions that support sustainable AI use among medical imaging workforces. Several studies acknowledged the key dimensions of AI adoption in radiology, including perceived benefit, trust, and attitudes toward AI adoption; however, these constructs have rarely been conceptualized within broader postadoption workforce adaptation processes, indicating a significant research gap in the literature.

This review contributes a conceptual framework that organizes the themes relating to adoption, adaptation behaviors, and workforce sustainability reported in the included literature, intended as a basis for future empirical testing. The review findings show that AI adoption has been examined extensively in terms of perceived benefits, trust, attitudes, and professional concerns, while evidence on postadoption adaptation and longer-term workforce sustainability remains comparatively limited. Thus, this imbalance leaves limited evidence on the organizational processes needed to protect radiologists’ professional interests, support well-being, and facilitate skills development during longer-term AI adaptation. This review offers a conceptual starting point for future research and organizational practice seeking to better understand and support workforce adaptation to AI in medical imaging.

## Supplementary material

10.2196/101944 Multimedia Appendix 1 Supplementary tables detailing database search strategies, characteristics of the 69 included studies, and criterion-level methodological quality assessments using the Mixed Methods Appraisal Tool (MMAT).

10.2196/101944 Checklist 1 PRISMA (Preferred Reporting Items for Systematic Reviews and Meta-Analyses) checklist.

## Abbreviations

CMUA: coping model of user adaptation
CT: computed tomography
EIT: electrical impedance tomography
HR: human resource
MMAT: Mixed Methods Appraisal Tool
MRI: magnetic resonance imaging
NASSS: Nonadoption, Abandonment, Scale-up, Spread, and Sustainability
PET: positron emission tomography
PRISMA: Preferred Reporting Items for Systematic Reviews and Meta-Analyses
PROSPERO: International Prospective Register of Systematic Reviews
SEIPS: Systems Engineering Initiative for Patient Safety
SPECT: single-photon emission computed tomography
TAM: Technology Acceptance Model
