# Supplementary material for: AI Adoption, Adaptation, and Workforce Sustainability in Medical Imaging: Systematic Review and Conceptual Framework
Source: JMIR AI. 2026 Nov 3;5:e101944. doi: 10.2196/101944 (PMC13730511; doi:10.2196/101944)
Supplement: Supplementary file 2 [file ai-v5-e101944-s001.docx]

**Appendix S1**

***Table S1****. Full search strings applied for each of the seven databases searched*

| Database | Search String |
| --- | --- |
| 1. Embase | ('artificial intelligence'/exp OR 'machine learning'/exp OR 'deep learning'/exp OR 'neural network'/exp) AND ('radiology'/exp OR radiolog*:ti,ab OR ct:ti,ab OR mri:ti,ab OR ultrasound:ti,ab) AND ('adaptation'/exp OR 'technology acceptance' OR 'professional role'/exp OR 'job satisfaction'/exp OR 'burnout'/exp OR 'workload'/exp OR 'work engagement'/exp OR 'occupational stress'/exp OR 'training'/exp OR adapt*:ti,ab OR accept*:ti,ab OR trust:ti,ab OR perception*:ti,ab OR attitude*:ti,ab OR expectation*:ti,ab OR satisf*:ti,ab OR workload:ti,ab OR burnout:ti,ab OR stress:ti,ab OR wellbeing:ti,ab OR 'well-being':ti,ab OR resist*:ti,ab OR 'role change':ti,ab OR 'professional identity':ti,ab OR training:ti,ab OR skill*:ti,ab) |
| 2. Scopus | (TITLE-ABS-KEY("artificial intelligence" OR AI OR "machine learning" OR "deep learning" OR "neural network*") AND TITLE-ABS-KEY(radiolog* OR "medical imaging professional*") AND TITLE-ABS-KEY( adapt* OR accept* OR trust OR expectation* OR satisf* OR burnout OR workload OR "role change" OR "role transformation" OR "professional identity" OR resist* OR "work engagement" OR training OR skill* OR "human-ai collaboration" OR "technology acceptance" OR perception* OR attitude* OR stress OR wellbeing OR "well-being" OR experience* OR "job satisfaction" OR "professional development" OR "career development" OR "occupational stress" OR workforce OR staffing OR retention OR recruitment OR "professional practice" OR "clinical practice" OR "clinical decision making" OR clinician* OR physician* OR "medical professional*" OR "health professional*" OR "healthcare professional*" OR "health care professional*" OR anxiety OR fear OR confidence OR competence OR competency OR "self-efficacy" OR autonomy OR empowerment ) ) |
| 3. Web of Science | ( ( ("artificial intelligence" OR AI OR "machine learning" OR "neural network*") AND (radiolog* OR "medical imaging department" OR "imaging department") AND ( adapt OR trust OR expectation* OR satisf* OR burnout OR workload OR "role change" OR "role transformation" OR "professional identity" OR resist* OR "work engagement" OR training OR skill* OR "human-ai collaboration" OR "technology acceptance" OR "job satisfaction" ) )) |
| 4. CINAHL | ((MH "Artificial Intelligence+"  OR MH "Machine Learning+"  OR TI ("artificial intelligence" OR AI OR "machine learning" OR "deep learning" OR "neural network*")  OR AB ("artificial intelligence" OR AI OR "machine learning" OR "deep learning" OR "neural network*"))  AND  (MH "Radiologists+"  OR MH "Radiology+"  OR MH "Medical Imaging+"  OR MH "Radiologic Technologists+"  OR MH "Imaging Services+"  OR TI (radiolog* OR "medical imaging")  OR AB (radiolog* OR "medical imaging"))  AND  (MH "Technology Acceptance+"  OR MH "Professional Role+"  OR MH "Job Satisfaction+"  OR MH "Burnout, Professional+"  OR MH "Workload+"  OR MH "Health Personnel+"  OR MH "Health Care Personnel+"  OR MH "Professional Practice+"  OR MH "Attitude to Computers+"  OR MH "Occupational Stress+"  OR MH "Work Environment+"  OR MH "Organizational Culture+"  OR MH "Personnel Staffing and Scheduling+"  OR MH "Workforce+"  OR MH "Allied Health Personnel+"  OR MH "Interdisciplinary Communication+"  OR MH "Interprofessional Relations+"  OR MH "Clinical Competence+"  OR MH "Professional Autonomy+"  OR MH "Decision Making+"  OR MH "Employee Attitudes+"  OR MH "Self Efficacy+"  OR TI (adapt* OR accept* OR trust OR expectation*  OR satisf* OR burnout OR workload  OR "role change" OR "role transformation"  OR "professional identity"  OR resist* OR "work engagement"  OR training OR skill*  OR "human-ai collaboration"  OR "technology acceptance"  OR "job satisfaction"  OR perception* OR attitude* OR experience*  OR stress OR wellbeing OR "well-being"  OR "clinical decision making"  OR clinician* OR physician*  OR "clinical workflow"  OR implementation  OR integration  OR deployment  OR "practice change"  OR autonomy OR confidence OR anxiety OR uncertainty OR "decision-making"  OR readiness OR preparedness OR "self-efficacy"  OR competence OR competency OR fear)  OR AB (adapt* OR accept* OR trust OR expectation*  OR satisf* OR burnout OR workload  OR "role change" OR "role transformation"  OR "professional identity"  OR resist* OR "work engagement"  OR training OR skill*  OR "human-ai collaboration"  OR "technology acceptance"  OR "job satisfaction"  OR perception* OR attitude* OR experience*  OR stress OR wellbeing OR "well-being"  OR "clinical decision making"  OR clinician* OR physician*  OR "clinical workflow"  OR implementation  OR integration  OR deployment  OR "practice change"  OR autonomy OR confidence OR anxiety OR uncertainty OR "decision-making"  OR readiness OR preparedness OR "self-efficacy"  OR competence OR competency OR fear OR engagement OR morale  OR coping  OR support  OR supervision  OR mentoring  OR teamwork))) |
| 5. ScienceDirect | ("artificial intelligence" OR "machine learning" OR "deep learning") AND radiology AND (adaptation OR job satisfaction OR performance) |
| 6. Medline (OVID) | ((artificial intelligence or AI or machine learning or deep learning or neural network*) and (radiolog* or radiologist*)).ti. and (adapt* or accept* or trust or "role change" or "role transformation" or "professional identity" or burnout or workload or "job satisfaction" or resist* or "technology acceptance" or implementation or integration or adoption or readiness or perception* or attitude* or "decision support" or workflow or satisfaction or experience* or perspective* or impact or effect* or evaluation or preparedness or competence or "clinical practice" or barrier* or facilitator* or challenge* or confidence or anxiety or usability or fear or engagement or collaboration or autonomy or "self-efficacy" or leadership or morale or "organizational culture").ti,ab. |
| 7. PsycINFO | (artificial intelligence or AI or machine learning or deep learning or neural network*).ti. and (radiolog* or radiologist*).ti,ab. and (adapt* or accept* or trust or "role change" or "role transformation" or "professional identity" or burnout or workload or "job satisfaction" or resist* or "technology acceptance" or implementation or integration or adoption or readiness or perception* or attitude* or "decision support" or workflow or satisfaction or experience* or perspective* or impact or effect* or evaluation or preparedness or competence or "clinical practice" or barrier* or facilitator* or challenge* or confidence or anxiety or usability or fear or engagement or collaboration or autonomy or "self-efficacy" or leadership or morale or "organizational culture").ti,ab. |

**Note.** Searches were conducted iteratively between 25 August and 1 September 2025. MH = subject heading (CINAHL); TI = title field; AB = abstract field; ti,ab = title and abstract fields (Ovid syntax); /exp = exploded subject heading (Embase); * = truncation (e.g., "adapt*" retrieves adapt, adapted, adaptation). ScienceDirect's syntax supports fewer Boolean terms, so a smaller, representative term set was used to keep results relevant and manageable.

**Appendix S2**

**Table S2.** *Characteristics of the included studies*

| ***ID*** | ***Authors*** | ***Year*** | ***Journal*** | ***Country*** | ***Method*** | ***Sample size^A^*** | ***Participants^B^*** | ***Radiology services and clinical focus^C^*** | ***Modality^D^*** | ***Theory, Model or framework*** | ***Workforce attitudes*** | ***HR and workforce sustainability*** | |
| --- | --- | --- | --- | --- | --- | --- | --- | --- | --- | --- | --- | --- | --- |
| 1 | Federico, et al. [1] | 2025 | Radiography | Italy | Quantitative | ≤10 | 2 | 7 | 1 | No theory | Investigated | Investigated | |
| 2 | Abuzaid, et al. [2] | 2022 | Academic Radiology | United Arab Emirates | Quantitative | 151–400 | 5 | 1 | 2 | No theory | Investigated | Investigated | |
| 3 | Aldhafeeri [3] | 2024 | BMC Medical Ethics | Saudi Arabia | Mixed | 151–400 | 2 | 1 | 2 | No theory | Investigated | Investigated | |
| 4 | Aldhafeeri [4] | 2022 | Insights into Imaging | Saudi Arabia | Quantitative | 401–1,000 | 2 | 1 | 3 | No theory | Investigated | Investigated | |
| 5 | Allam, et al. [5] | 2024 | European Radiology | Multinational study (three or more countries) | Quantitative | >1,000 | 4 | 1 | 2 | No theory | Investigated | Investigated | |
| 6 | Al Mohammad, et al. [6] | 2024 | British Journal of Radiology | Jordan | Quantitative | 151–400 | 5 | 1 | 3 | No theory | Investigated | Investigated | |
| 7 | Botwe, et al. [7] | 2021 | Radiography | Multinational study | Quantitative | >1,000 | 2 | 1 | 4 | No theory | Investigated | Investigated | |
| 8 | Buchlak, et al. [8] | 2024 | European Radiology | Two countries | Quantitative | 51–150 | 1 | 10 | 6 | No theory | Investigated | Investigated | |
| 9 | Cè, et al. [9] | 2024 | European Journal of Radiology | Italy | Quantitative | 151–400 | 1 | 1 | 3 | No theory | Investigated | Investigated | |
| 10 | Chen, et al. [10] | 2023 | Journal of Medical Internet Research | China | Quantitative | >1,000 | 3 | 1 | 1 | Mentioned but not applied | Investigated | Investigated | |
| 11 | Chen, et al. [11] | 2021 | BMC health services research | UK | Qualitative | 21–50 | 5 | 2 | 1 | Applied | Investigated | Investigated | |
| 12 | Coakley, et al. [12] | 2022 | Radiography | Multinational study | Quantitative | 51–150 | 2 | 1 | 3 | No theory | Investigated | Investigated | |
| 13 | Coppola, et al. [13] | 2021 | La radiologia medica | Italy | Quantitative | >1,000 | 1 | 1 | 2 | No theory | Investigated | Investigated | |
| 14 | Cotena, et al. [14] | 2024 | Diagnostics | United States | Quantitative | ≤10 | 1 | 8 | 6 | No theory | Not Investigated | Investigated | |
| 15 | Dratsch, et al. [15] | 2023 | Radiology | Germany | Quantitative | 21–50 | 1 | 2 | 5 | No theory | Investigated | Investigated | |
| 16 | Elhakim, et al. [16] | 2023 | Cancer Imaging | Denmark | Quantitative | 21–50 | 1 | 2 | 5 | No theory | Not Investigated | Investigated | |
| 17 | Eltorai, et al. [17] | 2020 | Journal of Thoracic Imaging | United States | Quantitative | 51–150 | 5 | 3 | 1 | No theory | Investigated | Investigated | |
| 18 | Fu, et al. [18] | 2023 | Radiology | China | Quantitative | 11–20 | 5 | 4 | 6 | No theory | Not Investigated | Investigated | |
| 19 | Gulum, et al. [19] | 2024 | Applied Sciences | Not explicitly stated / open-source / public domain | Mixed | ≤10 | 1 | 5 | 7 | No theory | Investigated | Investigated | |
| 20 | Guo, et al. [20] | 2024 | Frontiers in Neurology | China | Quantitative | 11–20 | 5 | 4 | 6 | No theory | Investigated | Investigated | |
| 21 | Hennrich, et al. [21] | 2025 | Journal of Medical Internet Research | Multinational study | Qualitative | 11–20 | 5 | 1 | 2 | Mentioned but not applied | Investigated | Investigated | |
| 22 | Hernström, et al. [22] | 2025 | The Lancet Digital Health | Sweden | Quantitative | 11–20 | 1 | 2 | 5 | No theory | Investigated | Investigated | |
| 23 | Hesso, et al. [23] | 2023 | Radiation Oncology | Multinational study | Mixed | 51–150 | 5 | 9 | 3 | Mentioned but not applied | Investigated | Investigated | |
| 24 | Högberg, et al. [24] | 2024 | BMJ Health and Care Informatics | Sweden | Mixed | 21–50 | 1 | 2 | 5 | No theory | Investigated | Investigated | |
| 25 | Hong, et al. [25] | 2025 | American Journal of Roentgenology | Two countries | Quantitative | ≤10 | 1 | 3 | 4 | No theory | Investigated | Investigated | |
| 26 | Hsieh [26] | 2023 | Computers in Human Behavior | Taiwan | Quantitative | 151–400 | 3 | 10 | 7 | Applied | Investigated | Investigated | |
| 27 | Huang, et al. [27] | 2025 | JAMA Network Open | United States | Quantitative | 21–50 | 1 | 10 | 4 | No theory | Investigated | Investigated | |
| 28 | Kim, et al. [28] | 2024 | Insights into Imaging | Netherlands | Qualitative | 11–20 | 5 | 10 | 3 | No theory | Investigated | Investigated | |
| 29 | Huisman, et al. [29] | 2021 | European Radiology | Multinational study | Quantitative | >1,000 | 5 | 10 | 1 | No theory | Investigated | Investigated | |
| 30 | Huisman, et al. [30] | 2021 | European Radiology | Multinational study | Quantitative | >1,000 | 5 | 10 | 1 | No theory | Investigated | Investigated | |
| 31 | Kim, et al. [31] | 2025 | Diagnostic and Interventional Radiology | South Korea | Quantitative | ≤10 | 1 | 4 | 6 | No theory | Not Investigated | Investigated | |
| 32 | Kim, et al. [32] | 2025 | La radiologia medica | Germany | Mixed | ≤10 | 5 | 4 | 7 | No theory | Investigated | Investigated | |
| 33 | Kim, et al. [33] | 2025 | European Radiology | Germany | Mixed | ≤10 | 3 | 4 | 7 | No theory | Investigated | Investigated | |
| 34 | Lång, et al. [34] | 2023 | The Lancet Oncology | Sweden | Quantitative | 11–20 | 1 | 2 | 5 | No theory | Investigated | Investigated | |
| 35 | Lee, et al. [35] | 2025 | The Spine Journal | Singapore | Quantitative | ≤10 | 5 | 6 | 7 | No theory | Investigated | Investigated | |
| 36 | Lee, et al. [36] | 2022 | Korean Journal of Radiology | South Korea | Quantitative | ≤10 | 1 | 2 | 5 | No theory | Investigated | Investigated | |
| 37 | Lee, et al. [37] | 2022 | Ultrasonography | South Korea | Quantitative | ≤10 | 5 | 2 | 8 | No theory | Not Investigated | Investigated | |
| 38 | Liu, et al. [38] | 2024 | JAMA Network Open | China | Quantitative | >1,000 | 1 | 1 | 3 | Applied | Investigated | Investigated | |
| 39 | Massey, et al. [39] | 2023 | American Journal of Otolaryngology | United States | Mixed | 51–150 | 3 | 4 | 6 | No theory | Investigated | Not Investigated | |
| 40 | Park, et al. [40] | 2024 | Radiology: Artificial Intelligence | Two countries | Quantitative | 11–20 | 1 | 2 | 5 | No theory | Investigated | Investigated | |
| 41 | Pedersen, et al. [41] | 2024 | Radiography | Multinational study | Mixed | 401–1,000 | 2 | 1 | 1 | Applied | Investigated | Investigated | |
| 42 | Perez, et al. [42] | 2024 | Journal of Vocational Behavior | France | Qualitative | 51–150 | 5 | 10 | 3 | Applied | Investigated | Investigated | |
| 43 | Peters, et al. [43] | 2024 | European Journal of Radiology | Germany | Quantitative | ≤10 | 5 | 4 | 7 | No theory | Not Investigated | Investigated | |
| 44 | Prinster, et al. [44] | 2024 | Radiology | United States | Quantitative | 151–400 | 5 | 3 | 4 | No theory | Investigated | Investigated | |
| 45 | Rainey, et al. [45] | 2022 | Radiography | UK | Quantitative | 401–1,000 | 2 | 10 | 3 | No theory | Investigated | Not Investigated | |
| 46 | Rainey, et al. [46] | 2021 | Frontiers in Digital Health | UK | Mixed | 401–1,000 | 2 | 10 | 3 | No theory | Investigated | Investigated | |
| 47 | Rasche, et al. [47] | 2024 | Radiography | Multinational study | Mixed | Not Reported | 2 | 10 | 4 | No theory | Not Investigated | Investigated | |
| 48 | Reschke, et al. [48] | 2025 | Academic Radiology | Germany | Quantitative | ≤10 | 1 | 6 | 7 | No theory | Not Investigated | Investigated | |
| 49 | Reschke, et al. [49] | 2025 | Academic Radiology | Germany | Quantitative | ≤10 | 1 | 5 | 7 | No theory | Not Investigated | Investigated | |
| 50 | Robert, et al. [50] | 2025 | Academic Radiology | United States | Quantitative | 11–20 | 5 | 3 | 4 | No theory | Not Investigated | Investigated | |
| 51 | Salim, et al. [51] | 2023 | British Journal of Radiology | Sweden | Quantitative | 21–50 | 1 | 2 | 5 | No theory | Not Investigated | Investigated | |
| 52 | Stogiannos, et al. [52] | 2024 | Radiography | UK | Mixed | 51–150 | 2 | 1 | 1 | No theory | Investigated | Investigated | |
| 53 | Stogiannos, et al. [53] | 2025 | Insights into Imaging | Multinational study | Quantitative | >1,000 | 2 | 1 | 1 | No theory | Investigated | Investigated | |
| 54 | Strohm, et al. [54] | 2020 | European Radiology | Netherlands | Qualitative | 21–50 | 5 | 10 | 4 | Applied | Investigated | Not Investigated | |
| 55 | Sun, et al. [55] | 2025 | PLoS ONE | China | Quantitative | 11–20 | 1 | 3 | 6 | No theory | Not Investigated | Investigated | |
| 56 | Tam, et al. [56] | 2021 | Clinical Radiology | UK | Quantitative | ≤10 | 1 | 3 | 4 | No theory | Investigated | Investigated | |
| 57 | Tang, et al. [57] | 2024 | Preventive Medicine Reports | Australia | Mixed | 51–150 | 5 | 2 | 5 | No theory | Investigated | Investigated | |
| 58 | Tong, et al. [58] | 2022 | BMC Medicine | China | Quantitative | 11–20 | 1 | 5 | 8 | No theory | Not Investigated | Investigated | |
| 59 | Tong, et al. [59] | 2023 | JAMA Network Open | China | Quantitative | 11–20 | 1 | 4 | 8 | No theory | Not Investigated | Investigated | |
| 60 | Wang, et al. [60] | 2022 | European Radiology | China | Quantitative | ≤10 | 1 | 2 | 7 | No theory | Not Investigated | Investigated | |
| 61 | Wang, et al. [61] | 2025 | Academic Radiology | China | Quantitative | ≤10 | 1 | 4 | 6 | No theory | Not Investigated | Investigated | |
| 62 | Wan, et al. [62] | 2025 | European Journal of Radiology | China | Quantitative | ≤10 | 5 | 6 | 6 | No theory | Not Investigated | Investigated | |
| 63 | Wenderott, et al. [63] | 2024 | Applied ergonomics | Germany | Qualitative | 11–20 | 5 | 5 | 7 | Applied | Investigated | Investigated | |
| 64 | Xia, et al. [64] | 2025 | Insights into Imaging | China | Quantitative | >1,000 | 5 | 1 | 1 | No theory | Investigated | Investigated | |
| 65 | Xing, et al. [65] | 2025 | Academic Radiology | China | Quantitative | ≤10 | 1 | 5 | 7 | No theory | Not Investigated | Investigated | |
| 66 | Yacoub, et al. [66] | 2022 | American Journal of Roentgenology | United States | Quantitative | ≤10 | 1 | 3 | 6 | No theory | Not Investigated | Investigated | |
| 67 | Yan, et al. [67] | 2024 | Communications Medicine | China | Quantitative | ≤10 | 1 | 2 | 8 | No theory | Investigated | Investigated | |
| 68 | Yoo, et al. [68] | 2022 | Korean Journal of Radiology | South Korea | Quantitative | 11–20 | 5 | 3 | 4 | No theory | Not Investigated | Investigated | |
| 69 | Zhu, et al. [69] | 2022 | BMC Medicine | China | Quantitative | ≤10 | 1 | 4 | 2 | No theory | Not Investigated | Investigated | |
| **Note**. **A**: If, in the included study, patient data were used solely as AI model input without collecting patient attitudes or insights on AI use or assistance, we did not include them in the total sample size;  **B**: 1=Radiologists, 2=Radiographers, 3=Other professionals and expertise (e.g. nurses, residents, interns, physicians, technicians, technologists, scientists), 4=Medical students, 5=Mixed participant study (two or more types of participants);  **C**: 1= Radiology in general (no specific services), 2=Breast Imaging, 3=Chest & Thoracic Radiology, 4=Neuroradiology & Head/Neck Imaging, 5= Abdominal & Pelvic Imaging, 6= Musculoskeletal Radiology, 7= Interventional Radiology, 8=Emergency Radiology, 9=Oncology Imaging, 10= Multiple services;  **D**: 1=Not Discussed/ Not Applicable), 2=Medical imaging in general, 3=Multi-modality (two or more modalities), 4=X-ray (including Radiograph, Fluoroscopy, DEXA), 5=Mammography (including Digital Breast Tomosynthesis), 6=CT, 7=MRI, 8= Ultrasound / ultrasonography | | | | | | | | | | | | |  |

**Appendix S3**

**Table S3.** *MMAT criterion-level appraisal of qualitative studies*

| ***ID*** | ***Author(s)*** | ***Year*** | ***S1. Are there clear research aims/questions?*** | ***S2. Do the collected data allow to address the research questions?*** | ***1.1. Is the qualitative approach appropriate to answer the research question?*** | ***1.2. Are the qualitative data collection methods adequate to address the research question?*** | ***1.3. Are the findings adequately derived from the data?*** | ***1.4. Is the interpretation of results sufficiently substantiated by data?*** | ***1.5. Is there coherence between qualitative data sources, collection, analysis and interpretation?*** |
| --- | --- | --- | --- | --- | --- | --- | --- | --- | --- |
| 1 | *Chen, Stavropoulou, Narasinkan, Baker and Scarbrough [11]* | 2021 | Yes | Yes | Yes | Yes | Yes | Yes | Yes |
| 2 | *Hennrich, Doctor, Körner, Lederman and Eymann [21]* | 2025 | Yes | Yes | Yes | Yes | Yes | Yes | Yes |
| 3 | *Kim, Romeijn, van Buchem, Mehrizi and Grootjans [28]* | 2024 | Yes | Yes | Yes | Yes | Yes | Yes | Yes |
| 4 | *Perez, Conway, Peterson and Roques [42]* | 2024 | Yes | Yes | Yes | Yes | Yes | Yes | Yes |
| 5 | *Strohm, Hehakaya, Ranschaert, Boon and Moors [54]* | 2020 | Yes | Yes | Yes | Yes | Yes | Can’t tell | Yes |
| 6 | *Wenderott, Krups, Luetkens and Weigl [63]* | 2024 | Yes | Yes | Yes | Yes | Yes | Yes | Yes |

**Table S4.** *MMAT criterion-level appraisal of quantitative studies*

| ***ID*** | ***Author(s)*** | ***Year*** | ***S1. Are there clear research aims/questions?*** | ***S2. Do the collected data allow to address the research questions?*** | ***4.1. Is the sampling strategy relevant to address the research question?*** | ***4.2. Is the sample representative of the target population?*** | ***4.3. Are the measurements appropriate?*** | ***4.4. Is the risk of nonresponse bias low?*** | ***4.5. Is the statistical analysis appropriate to answer the research question?*** |
| --- | --- | --- | --- | --- | --- | --- | --- | --- | --- |
| 7 | *Federico, Fusaro, Coppola, Gregori and Durante [1]* | 2025 | Yes | Yes | Yes | No | Yes | Can’t tell | Yes |
| 8 | *Abuzaid, Elshami, Tekin and Issa [2]* | 2022 | Yes | Yes | Yes | Can’t tell | Yes | Can’t tell | Yes |
| 9 | *Aldhafeeri [4]* | 2022 | Yes | Yes | Yes | Yes | Yes | Can’t tell | Yes |
| 10 | *Allam, Eltewacy, Alabdallat, Owais, Salman and Ebada [5]* | 2024 | Yes | Yes | Yes | Can’t tell | Yes | Can’t tell | Yes |
| 11 | *Al Mohammad, Aldaradkeh, Gharaibeh and Reed [6]* | 2024 | Yes | Yes | Yes | No | Yes | Yes | Yes |
| 12 | *Botwe, Akudjedu, Antwi, Rockson, Mkoloma, Balogun, Elshami, Bwambale, Barare and Mdletshe [7]* | 2021 | Yes | Yes | Yes | No | Yes | Can’t tell | Yes |
| 13 | *Buchlak, Tang, Seah, Johnson, Holt, Bottrell, Wardman, Samarasinghe, Dos Santos Pinheiro and Xia [8]* | 2024 | Yes | Yes | Yes | No | Yes | Yes | Yes |
| 14 | *Cè, Ibba, Cellina, Tancredi, Fantesini, Fazzini, Fortunati, Perazzo, Presta and Montanari [9]* | 2024 | Yes | Yes | Yes | No | Yes | No | Yes |
| 15 | *Chen, Wu, Wang, Xie, Yan, Jiang, Yang, Zheng, Zhang and Zhu [10]* | 2023 | Yes | Yes | Yes | Yes | Yes | No | Yes |
| 16 | *Coakley, Young, Moore, England, O'Mahony, O'Connor, Maher and McEntee [12]* | 2022 | Yes | Yes | Yes | No | Yes | No | Yes |
| 17 | *Coppola, Faggioni, Regge, Giovagnoni, Golfieri, Bibbolino, Miele, Neri and Grassi [13]* | 2021 | Yes | Yes | Yes | No | Yes | Can’t tell | Yes |
| 18 | *Cotena, Ayobi, Zuchowski, Junn, Weinberg, Chang, Chow, Soun, Roca-Sogorb and Chaibi [14]* | 2024 | Yes | Yes | Yes | Yes | Yes | Yes | Yes |
| 19 | *Dratsch, Chen, Rezazade Mehrizi, Kloeckner, Mähringer-Kunz, Püsken, Baeßler, Sauer, Maintz and Pinto dos Santos [15]* | 2023 | Yes | Yes | Yes | Can’t tell | Yes | Can’t tell | Yes |
| 20 | *Elhakim, Stougaard, Graumann, Nielsen, Lång, Gerke, Larsen and Rasmussen [16]* | 2023 | Yes | Yes | Yes | Yes | Yes | Yes | Yes |
| 21 | *Eltorai, Bratt and Guo [17]* | 2020 | Yes | Yes | Yes | No | Yes | No | Yes |
| 22 | *Fu, Shan, Yang, Zheng, Zhang, Rong, Wang and Lu [18]* | 2023 | Yes | Yes | Yes | Yes | Yes | Can’t tell | Yes |
| 23 | *Guo, Liu, Xu, Huang, Zhang, Miao, Zhang, Li, Shan and Gu [20]* | 2024 | Yes | Yes | Yes | No | Yes | Can’t tell | Yes |
| 24 | *Hernström, Josefsson, Sartor, Schmidt, Larsson, Hofvind, Andersson, Rosso, Hagberg and Lång [22]* | 2025 | Yes | Yes | Yes | Yes | Yes | Yes | Yes |
| 25 | *Hong, Kim, Song, Lee, Kim, Cho, Kim, Lee, Bae and Roh [25]* | 2025 | Yes | Yes | Yes | Yes | Yes | Yes | Yes |
| 26 | *Hsieh [26]* | 2023 | Yes | Yes | Yes | No | Yes | Yes | Yes |
| 27 | *Huang, Wittbrodt, Teague, Karl, Galal, Thompson, Chapa, Chiu, Herynk and Linchangco [27]* | 2025 | Yes | Yes | Yes | Yes | Yes | Yes | Yes |
| 28 | *Huisman, Ranschaert, Parker, Mastrodicasa, Koci, Pinto de Santos, Coppola, Morozov, Zins and Bohyn [29]* | 2021 | Yes | Yes | Yes | No | Yes | No | Yes |
| 29 | *Huisman, Ranschaert, Parker, Mastrodicasa, Koci, Pinto de Santos, Coppola, Morozov, Zins and Bohyn [30]* | 2021 | Yes | Yes | Yes | No | Yes | No | Yes |
| 30 | *Kim, Jang, Oh, Lee, Min, Choi and Ahn [31]* | 2025 | Yes | Yes | Yes | Yes | Yes | Yes | Yes |
| 31 | *Lång, Josefsson, Larsson, Larsson, Högberg, Sartor, Hofvind, Andersson and Rosso [34]* | 2023 | Yes | Yes | Yes | Yes | Yes | Yes | Yes |
| 32 | *Lee, Wu, Liu, Makmur, Ting, Lee, Chan, Lim, Khoo and Sng [35]* | 2025 | Yes | Yes | Yes | No | Yes | Can’t tell | Yes |
| 33 | *Lee, Kim, Lee, Ahn, Ryu, Park, Shin, Kim and Choi [36]* | 2022 | Yes | Yes | Yes | No | Yes | Yes | Yes |
| 34 | *Lee, Han, Youk, Lee, Hwang, Rho, Yoon, Kim and Yoon [37]* | 2022 | Yes | Yes | Yes | No | Yes | Can’t tell | Yes |
| 35 | *Liu, Ding, Li, Chen, Sun, Huang, Liu, Ye, Jin and Bao [38]* | 2024 | Yes | Yes | Yes | Yes | Yes | Yes | Yes |
| 36 | *Park, Kwak, Lee, Choi, Kooi and Kim [40]* | 2024 | Yes | Yes | Yes | Can’t tell | Yes | Can’t tell | Yes |
| 37 | *Peters, Kellermann, Watkinson, Gärtner, Huhndorf, Stürner, Jansen and Larsen [43]* | 2024 | Yes | Yes | Yes | Can’t tell | Yes | Can’t tell | Yes |
| 38 | *Prinster, Mahmood, Saria, Jeudy, Lin, Yi and Huang [44]* | 2024 | Yes | Yes | Yes | Can’t tell | Yes | Can’t tell | Yes |
| 39 | *Rainey, O'Regan, Matthew, Skelton, Woznitza, Chu, Goodman, McConnell, Hughes and Bond [45]* | 2022 | Yes | Yes | Yes | No | Yes | Can’t tell | Yes |
| 40 | *Reschke, Gotta, Gruenewald, Ait Bachir, Strecker, Nickel, Booz, Martin, Scholtz and D’Angelo [48]* | 2025 | Yes | Yes | Yes | No | Yes | Can’t tell | Yes |
| 41 | *Reschke, Koch, Gruenewald, Ait Bachir, Gotta, Booz, Alrahmoun, Strecker, Nickel and D’Angelo [49]* | 2025 | Yes | Yes | Yes | No | Yes | Can’t tell | Yes |
| 42 | *Robert, Sathyamurthy, Singh, Matta, Tadepalli, Tanamala, Bosemani, Mammarappallil and Kundnani [50]* | 2025 | Yes | Yes | Yes | Yes | Yes | Yes | Yes |
| 43 | *Salim, Dembrower, Eklund, Smith and Strand [51]* | 2023 | Yes | Yes | Yes | Yes | Yes | Can’t tell | Yes |
| 44 | *Stogiannos, Walsh, Ohene-Botwe, McHugh, Potts, Tam, O’Sullivan, Quinsten, Gibson and Gorga [53]* | 2025 | Yes | Yes | Yes | Yes | Yes | No | Yes |
| 45 | *Sun, Fan, Shi, Sun, Ma, Zhang, Zhang, Liu, Yu and Tong [55]* | 2025 | Yes | Yes | Yes | Yes | Yes | Yes | Yes |
| 46 | *Tam, Dyer, Dissez, Morgan, Hughes, Illes, Rasalingham and Rasalingham [56]* | 2021 | Yes | Yes | Yes | No | Yes | Yes | Yes |
| 47 | *Tong, Gu, Xu, Song, Zhao, Cheng, Yuan, Tian, Yang and Tian [58]* | 2022 | Yes | Yes | Yes | Yes | Yes | Yes | Yes |
| 48 | *Tong, Wu, Cheng, Huang, Liang, Li, Guo, He, Liu and Xiao [59]* | 2023 | Yes | Yes | Yes | Yes | Yes | Yes | Yes |
| 49 | *Wang, Chang, Luo, Cui, Liu, Wu, Chen, Zhang, Wu, Li, Liu, Guan and Wang [60]* | 2022 | Yes | Yes | Yes | Yes | Yes | Can’t tell | Yes |
| 50 | *Wang, Liu, Zhang, Wang, Yu, Wang, Zhang, Wang, Zhang, Yang, Mou, Mao and Song [61]* | 2025 | Yes | Yes | Yes | Yes | Yes | Can’t tell | Yes |
| 51 | *Wan, Su, Xiong, Cui, Tang, Zhang and Zhang [62]* | 2025 | Yes | Yes | Yes | Yes | Yes | Can’t tell | Yes |
| 52 | *Xia, Zhang, Zhao, Lei, Xiao, Chen, Zha, Yu, Wu and Lu [64]* | 2025 | Yes | Yes | Yes | Yes | Yes | Yes | Yes |
| 53 | *Xing, Chen, Pan, Huang, Qiu, Sheng, Zhang, Wang, Cheng and Xing [65]* | 2025 | Yes | Yes | Yes | Yes | Yes | Yes | Yes |
| 54 | *Yacoub, Varga-Szemes, Schoepf, Kabakus, Baruah, Burt, Aquino, Sullivan, Doherty and Hoelzer [66]* | 2022 | Yes | Yes | Yes | Yes | Yes | Yes | Yes |
| 55 | *Yan, Liang, Zhang, Zhang, Zheng, Han, Yu, Zhang, Xie and Liu [67]* | 2024 | Yes | Yes | Yes | No | Yes | Can’t tell | Yes |
| 56 | *Yoo, Kim, Kim, Choi, Kim, Hwang, Kim, Cho and Jin [68]* | 2022 | Yes | Yes | Yes | No | Yes | Can’t tell | Yes |
| 57 | *Zhu, Meng, Fan, Duan, Jia, Dong, Wang, Song, Tian and Wang [69]* | 2022 | Yes | Yes | Yes | No | Yes | Can’t tell | Yes |

**Table S5.** *. MMAT criterion-level appraisal of mixed-methods studies*

| ***ID*** | ***Author(s)*** | ***Year*** | ***S1. Are there clear research aims/questions?*** | ***S2. Do the collected data allow to address the research questions?*** | ***5.1. Is there an adequate rationale for using a mixed methods design to address the research question?*** | ***5.2. Are the different components of the study effectively integrated to answer the research question?*** | ***5.3. Are the outputs of the integration of qualitative and quantitative components adequately interpreted?*** | ***5.4. Are divergences and inconsistencies between quantitative and qualitative results adequately addressed?*** | ***5.5. Do the different components of the study adhere to the quality criteria of each tradition of the methods involved?*** |
| --- | --- | --- | --- | --- | --- | --- | --- | --- | --- |
| 58 | *Aldhafeeri [3]* | 2024 | Yes | Yes | Yes | Yes | Yes | Yes | No |
| 59 | *Gulum, Trombley, Ozen, Esen, Aksamoglu and Kantardzic [19]* | 2024 | Yes | Yes | Yes | Yes | Yes | Yes | No |
| 60 | *Hesso, Kayyali, Dolton, Joo, Zacharias, Charalambous, Lavdaniti, Stalika, Ajami and Acampa [23]* | 2023 | Yes | Yes | Yes | Yes | Yes | Yes | No |
| 61 | *Högberg, Larsson and Lång [24]* | 2024 | Yes | Yes | Yes | Yes | Yes | Yes | No |
| 62 | *Kim, Schramm, Riedel, Schmitzer, Rosenkranz, Kertels, Bodden, Paprottka, Sepp and Renz [32]* | 2025 | Yes | Yes | No | Yes | Yes | Yes | Can't tell |
| 63 | *Kim, Wihl, Schramm, Berberich, Rosenkranz, Schmitzer, Serguen, Klenk, Lenhart and Zimmer [33]* | 2025 | Yes | Yes | No | Yes | Yes | Yes | Can't tell |
| 64 | *Massey, Asokan, Tietbohl, Morris and Ramakrishnan [39]* | 2023 | Yes | Yes | Yes | Yes | Yes | Yes | No |
| 65 | *Pedersen, Kusk, Lysdahlgaard, Mork-Knudsen, Malamateniou and Jensen [41]* | 2024 | Yes | Yes | Yes | Yes | Yes | Yes | No |
| 66 | *Rainey, O'Regan, Matthew, Skelton, Woznitza, Chu, Goodman, McConnell, Hughes, Bond, McFadden and Malamateniou [46]* | 2021 | Yes | Yes | No | Yes | Yes | Yes | No |
| 67 | *Rasche, Brader, Borggrefe, Seuss, Carr, Hebecker and Ten Cate [47]* | 2024 | Yes | Yes | No | Yes | Yes | Yes | No |
| 68 | *Stogiannos, O'Regan, Scurr, Litosseliti, Pogose, Harvey, Kumar, Malik, Barnes and McEntee [52]* | 2024 | Yes | Yes | Can’t tell | Yes | Yes | Can’t tell | Yes |
| 69 | *Tang, Frazer, Kunicki, Basnayake, Omori and Lippey [57]* | 2024 | Yes | Yes | No | Yes | Yes | Yes | No |

**References**

[1] L. Federico, D. Fusaro, G. Coppola, M. Gregori, S. Durante, Application of ChatGPT 4.0 in radiological dose management: Perceptions of radiographers with varying expertise, Radiography 31(4) (2025) 102972.

[2] M.M. Abuzaid, W. Elshami, H. Tekin, B. Issa, Assessment of the willingness of radiologists and radiographers to accept the integration of artificial intelligence into radiology practice, Academic Radiology 29(1) (2022) 87-94.

[3] F.M. Aldhafeeri, Navigating the ethical landscape of artificial intelligence in radiography: a cross-sectional study of radiographers’ perspectives, BMC Medical Ethics 25(1) (2024) 52.

[4] F.M. Aldhafeeri, Perspectives of radiographers on the emergence of artificial intelligence in diagnostic imaging in Saudi Arabia, Insights into imaging 13(1) (2022) 178.

[5] A.H. Allam, N.K. Eltewacy, Y.J. Alabdallat, T.A. Owais, S. Salman, M.A. Ebada, Knowledge, attitude, and perception of Arab medical students towards artificial intelligence in medicine and radiology: a multi-national cross-sectional study, European radiology 34(7) (2024) 1-14.

[6] B. Al Mohammad, A. Aldaradkeh, M. Gharaibeh, W. Reed, Assessing radiologists’ and radiographers’ perceptions on artificial intelligence integration: opportunities and challenges, British Journal of Radiology 97(1156) (2024) 763-769.

[7] B. Botwe, T. Akudjedu, W. Antwi, P. Rockson, S. Mkoloma, E. Balogun, W. Elshami, J. Bwambale, C. Barare, S. Mdletshe, The integration of artificial intelligence in medical imaging practice: perspectives of African radiographers, Radiography 27(3) (2021) 861-866.

[8] Q.D. Buchlak, C.H. Tang, J.C. Seah, A. Johnson, X. Holt, G.M. Bottrell, J.B. Wardman, G. Samarasinghe, L. Dos Santos Pinheiro, H. Xia, Effects of a comprehensive brain computed tomography deep learning model on radiologist detection accuracy, European Radiology 34(2) (2024) 810-822.

[9] M. Cè, S. Ibba, M. Cellina, C. Tancredi, A. Fantesini, D. Fazzini, A. Fortunati, C. Perazzo, R. Presta, R. Montanari, Radiologists’ perceptions on AI integration: An in-depth survey study, European Journal of Radiology 177 (2024) 111590.

[10] Y. Chen, Z. Wu, P. Wang, L. Xie, M. Yan, M. Jiang, Z. Yang, J. Zheng, J. Zhang, J. Zhu, Radiology residents’ perceptions of artificial intelligence: nationwide cross-sectional survey study, Journal of Medical Internet Research 25 (2023) e48249.

[11] Y. Chen, C. Stavropoulou, R. Narasinkan, A. Baker, H. Scarbrough, Professionals’ responses to the introduction of AI innovations in radiology and their implications for future adoption: a qualitative study, BMC health services research 21(1) (2021) 813.

[12] S. Coakley, R. Young, N. Moore, A. England, A. O'Mahony, O.J. O'Connor, M. Maher, M.F. McEntee, Radiographers’ knowledge, attitudes and expectations of artificial intelligence in medical imaging, Radiography 28(4) (2022) 943-948.

[13] F. Coppola, L. Faggioni, D. Regge, A. Giovagnoni, R. Golfieri, C. Bibbolino, V. Miele, E. Neri, R. Grassi, Artificial intelligence: radiologists’ expectations and opinions gleaned from a nationwide online survey, La radiologia medica 126(1) (2021) 63-71.

[14] M. Cotena, A. Ayobi, C. Zuchowski, J.C. Junn, B.D. Weinberg, P.D. Chang, D.S. Chow, J.E. Soun, M. Roca-Sogorb, Y. Chaibi, Enhancing radiologist efficiency with AI: a multi-reader multi-case study on aortic dissection detection and prioritization, Diagnostics 14(23) (2024) 2689.

[15] T. Dratsch, X. Chen, M. Rezazade Mehrizi, R. Kloeckner, A. Mähringer-Kunz, M. Püsken, B. Baeßler, S. Sauer, D. Maintz, D. Pinto dos Santos, Automation bias in mammography: the impact of artificial intelligence BI-RADS suggestions on reader performance, Radiology 307(4) (2023) e222176.

[16] M.T. Elhakim, S.W. Stougaard, O. Graumann, M. Nielsen, K. Lång, O. Gerke, L.B. Larsen, B.S.B. Rasmussen, Breast cancer detection accuracy of AI in an entire screening population: a retrospective, multicentre study, Cancer Imaging 23(1) (2023) 127.

[17] A.E. Eltorai, A.K. Bratt, H.H. Guo, Thoracic radiologists’ versus computer scientists’ perspectives on the future of artificial intelligence in radiology, Journal of thoracic imaging 35(4) (2020) 255-259.

[18] F. Fu, Y. Shan, G. Yang, C. Zheng, M. Zhang, D. Rong, X. Wang, J. Lu, Deep learning for head and neck CT angiography: stenosis and plaque classification, Radiology 307(3) (2023) e220996.

[19] M.A. Gulum, C.M. Trombley, M. Ozen, E. Esen, M. Aksamoglu, M. Kantardzic, Why are explainable AI methods for prostate lesion detection rated poorly by radiologists?, Applied Sciences 14(11) (2024) 4654.

[20] Z. Guo, Y. Liu, J. Xu, C. Huang, F. Zhang, C. Miao, Y. Zhang, M. Li, H. Shan, Y. Gu, A deep learning model for carotid plaques detection based on CTA images: a two stepwise early-stage clinical validation study, Frontiers in Neurology 15 (2025) 1480792.

[21] J. Hennrich, E. Doctor, M.-F. Körner, R. Lederman, T. Eymann, Enabling Physicians to Make an Informed Adoption Decision on Artificial Intelligence Applications in Medical Imaging Diagnostics: Qualitative Study, Journal of Medical Internet Research 27 (2025) e63668.

[22] V. Hernström, V. Josefsson, H. Sartor, D. Schmidt, A.-M. Larsson, S. Hofvind, I. Andersson, A. Rosso, O. Hagberg, K. Lång, Screening performance and characteristics of breast cancer detected in the Mammography Screening with Artificial Intelligence trial (MASAI): a randomised, controlled, parallel-group, non-inferiority, single-blinded, screening accuracy study, The Lancet Digital Health 7(3) (2025) e175-e183.

[23] I. Hesso, R. Kayyali, D.-R. Dolton, K. Joo, L. Zacharias, A. Charalambous, M. Lavdaniti, E. Stalika, T. Ajami, W. Acampa, Cancer care at the time of the fourth industrial revolution: an insight to healthcare professionals’ perspectives on cancer care and artificial intelligence, Radiation Oncology 18(1) (2023) 167.

[24] C. Högberg, S. Larsson, K. Lång, Anticipating artificial intelligence in mammography screening: views of Swedish breast radiologists, BMJ Health & Care Informatics 30(1) (2023) e100712.

[25] E.K. Hong, H.W. Kim, O.K. Song, K.-C. Lee, D.K. Kim, J.-B. Cho, J. Kim, S. Lee, W. Bae, B. Roh, Multimodal Generative Artificial Intelligence Model for Creating Radiology Reports for Chest Radiographs in Patients Undergoing Tuberculosis Screening, American Journal of Roentgenology 225(4) (2025) e2533059.

[26] P.-J. Hsieh, Determinants of physicians’ intention to use AI-assisted diagnosis: An integrated readiness perspective, Computers in Human Behavior 147 (2023) 107868.

[27] J. Huang, M.T. Wittbrodt, C.N. Teague, E. Karl, G. Galal, M. Thompson, A. Chapa, M.-L. Chiu, B. Herynk, R. Linchangco, Efficiency and Quality of Generative AI–Assisted Radiograph Reporting, JAMA Network Open 8(6) (2025) e2513921-e2513921.

[28] B. Kim, S. Romeijn, M. van Buchem, M.H.R. Mehrizi, W. Grootjans, A holistic approach to implementing artificial intelligence in radiology, Insights Into Imaging 15(1) (2024) 22.

[29] M. Huisman, E. Ranschaert, W. Parker, D. Mastrodicasa, M. Koci, D. Pinto de Santos, F. Coppola, S. Morozov, M. Zins, C. Bohyn, An international survey on AI in radiology in 1,041 radiologists and radiology residents part 1: fear of replacement, knowledge, and attitude, European radiology 31(9) (2021) 7058-7066.

[30] M. Huisman, E. Ranschaert, W. Parker, D. Mastrodicasa, M. Koci, D. Pinto de Santos, F. Coppola, S. Morozov, M. Zins, C. Bohyn, An international survey on AI in radiology in 1041 radiologists and radiology residents part 2: expectations, hurdles to implementation, and education, European Radiology 31(11) (2021) 8797-8806.

[31] J. Kim, J. Jang, S.W. Oh, H.Y. Lee, E.J. Min, J.W. Choi, K.-J. Ahn, Impact of a computed tomography-based artificial intelligence software on radiologists’ workflow for detecting acute intracranial hemorrhage, Diagnostic and Interventional Radiology 31(5) (2025) 518.

[32] S.H. Kim, S. Schramm, E.O. Riedel, L. Schmitzer, E. Rosenkranz, O. Kertels, J. Bodden, K. Paprottka, D. Sepp, M. Renz, Automation bias in AI-assisted detection of cerebral aneurysms on time-of-flight MR angiography, La radiologia medica 130(4) (2025) 555-566.

[33] S.H. Kim, J. Wihl, S. Schramm, C. Berberich, E. Rosenkranz, L. Schmitzer, K. Serguen, C. Klenk, N. Lenhart, C. Zimmer, Human-AI collaboration in large language model-assisted brain MRI differential diagnosis: a usability study, European Radiology 35(9) (2025) 1-12.

[34] K. Lång, V. Josefsson, A.-M. Larsson, S. Larsson, C. Högberg, H. Sartor, S. Hofvind, I. Andersson, A. Rosso, Artificial intelligence-supported screen reading versus standard double reading in the Mammography Screening with Artificial Intelligence trial (MASAI): a clinical safety analysis of a randomised, controlled, non-inferiority, single-blinded, screening accuracy study, The Lancet Oncology 24(8) (2023) 936-944.

[35] A. Lee, J. Wu, C. Liu, A. Makmur, Y.H. Ting, S. Lee, M.D.Z. Chan, D.S.W. Lim, V.M.H. Khoo, J. Sng, Using deep learning to enhance reporting efficiency and accuracy in degenerative cervical spine MRI, The Spine Journal 25(9) (2025) 1942-1950.

[36] J.H. Lee, K.H. Kim, E.H. Lee, J.S. Ahn, J.K. Ryu, Y.M. Park, G.W. Shin, Y.J. Kim, H.Y. Choi, Improving the Performance of Radiologists Using Artificial Intelligence-Based Detection Support Software for Mammography: A Multi-Reader Study, Korean Journal of Radiology 23(5) (2022) 505-516.

[37] S.E. Lee, K. Han, J.H. Youk, J.E. Lee, J.-Y. Hwang, M. Rho, J. Yoon, E.-K. Kim, J.H. Yoon, Differing benefits of artificial intelligence-based computer-aided diagnosis for breast US according to workflow and experience level, Ultrasonography 41(4) (2022) 718-727.

[38] H. Liu, N. Ding, X. Li, Y. Chen, H. Sun, Y. Huang, C. Liu, P. Ye, Z. Jin, H. Bao, Artificial intelligence and radiologist burnout, JAMA network open 7(11) (2024) e2448714-e2448714.

[39] C.J. Massey, A. Asokan, C. Tietbohl, M. Morris, V.R. Ramakrishnan, Otolaryngologist perceptions of AI-based sinus CT interpretation, American Journal of Otolaryngology 44(5) (2023) 103932.

[40] E.K. Park, S. Kwak, W. Lee, J.S. Choi, T. Kooi, E.-K. Kim, Impact of AI for digital breast tomosynthesis on breast cancer detection and interpretation time, Radiology: Artificial Intelligence 6(3) (2024) e230318.

[41] M. Pedersen, M. Kusk, S. Lysdahlgaard, H. Mork-Knudsen, C. Malamateniou, J. Jensen, A Nordic survey on artificial intelligence in the radiography profession–is the profession ready for a culture change?, Radiography 30(4) (2024) 1106-1115.

[42] F. Perez, N. Conway, J. Peterson, O. Roques, Me, my work and AI: How radiologists craft their work and identity, Journal of Vocational Behavior 155 (2024) 104042.

[43] S. Peters, G. Kellermann, J. Watkinson, F. Gärtner, M. Huhndorf, K. Stürner, O. Jansen, N. Larsen, AI supported detection of cerebral multiple sclerosis lesions decreases radiologic reporting times, European Journal of Radiology 178 (2024) 111638.

[44] D. Prinster, A. Mahmood, S. Saria, J. Jeudy, C.T. Lin, P.H. Yi, C.-M. Huang, Care to explain? AI explanation types differentially impact chest radiograph diagnostic performance and physician trust in AI, Radiology 313(2) (2024) e233261.

[45] C. Rainey, T. O'Regan, J. Matthew, E. Skelton, N. Woznitza, K.-Y. Chu, S. Goodman, J. McConnell, C. Hughes, R. Bond, UK reporting radiographers’ perceptions of AI in radiographic image interpretation–Current perspectives and future developments, Radiography 28(4) (2022) 881-888.

[46] C. Rainey, T. O'Regan, J. Matthew, E. Skelton, N. Woznitza, K.-Y. Chu, S. Goodman, J. McConnell, C. Hughes, R. Bond, S. McFadden, C. Malamateniou, Beauty Is in the AI of the Beholder: Are We Ready for the Clinical Integration of Artificial Intelligence in Radiography? An Exploratory Analysis of Perceived AI Knowledge, Skills, Confidence, and Education Perspectives of UK Radiographers, Frontiers in digital health 3 (2021) 739327.

[47] A. Rasche, P. Brader, J. Borggrefe, H. Seuss, Z. Carr, A. Hebecker, G. Ten Cate, Impact of intelligent virtual and AI-based automated collimation functionalities on the efficiency of radiographic acquisitions, Radiography 30(4) (2024) 1073-1079.

[48] P. Reschke, J. Gotta, L.D. Gruenewald, A. Ait Bachir, R. Strecker, D. Nickel, C. Booz, S.S. Martin, J.-E. Scholtz, T. D’Angelo, Deep learning in knee mri: A prospective study to enhance efficiency, diagnostic confidence and sustainability, Academic Radiology 32(6) (2025) 3585-3596.

[49] P. Reschke, V. Koch, L.D. Gruenewald, A. Ait Bachir, J. Gotta, C. Booz, M.A. Alrahmoun, R. Strecker, D. Nickel, T. D’Angelo, Deep learning-accelerated prostate MRI: improving speed, accuracy, and sustainability, Academic Radiology 32(11) (2025) 6718-6728.

[50] D. Robert, S. Sathyamurthy, A.K. Singh, S.A. Matta, M. Tadepalli, S. Tanamala, V. Bosemani, J. Mammarappallil, B. Kundnani, Effect of artificial intelligence as a second reader on the lung nodule detection and localization accuracy of radiologists and non-radiology physicians in chest radiographs: a multicenter reader study, Academic Radiology 32(3) (2025) 1706-1717.

[51] M. Salim, K. Dembrower, M. Eklund, K. Smith, F. Strand, Differences and similarities in false interpretations by AI CAD and radiologists in screening mammography, The British Journal of Radiology 96(1151) (2023) 20230210.

[52] N. Stogiannos, T. O'Regan, E. Scurr, L. Litosseliti, M. Pogose, H. Harvey, A. Kumar, R. Malik, A. Barnes, M. McEntee, AI implementation in the UK landscape: knowledge of AI governance, perceived challenges and opportunities, and ways forward for radiographers, Radiography 30(2) (2024) 612-621.

[53] N. Stogiannos, G. Walsh, B. Ohene-Botwe, K. McHugh, B. Potts, W. Tam, C. O’Sullivan, A.S. Quinsten, C. Gibson, R.G. Gorga, R-AI-diographers: a European survey on perceived impact of AI on professional identity, careers, and radiographers’ roles, Insights into imaging 16(1) (2025) 43.

[54] L. Strohm, C. Hehakaya, E.R. Ranschaert, W.P. Boon, E.H. Moors, Implementation of artificial intelligence (AI) applications in radiology: hindering and facilitating factors, European radiology 30(10) (2020) 5525-5532.

[55] L. Sun, Y. Fan, S. Shi, M. Sun, Y. Ma, K. Zhang, F. Zhang, H. Liu, T. Yu, H. Tong, AI-assisted radiologists vs. standard double reading for rib fracture detection on CT images: A real-world clinical study, PloS one 20(1) (2025) e0316732.

[56] M. Tam, T. Dyer, G. Dissez, T.N. Morgan, M. Hughes, J. Illes, R. Rasalingham, S. Rasalingham, Augmenting lung cancer diagnosis on chest radiographs: Positioning artificial intelligence to improve radiologist performance, Clinical Radiology 76(8) (2021) 607-614.

[57] J.S. Tang, H.M. Frazer, K. Kunicki, P. Basnayake, M. Omori, J. Lippey, Australian healthcare workers’ views on artificial intelligence in BreastScreen: Results of a mixed method survey study, Preventive Medicine Reports 48 (2024) 102917.

[58] T. Tong, J. Gu, D. Xu, L. Song, Q. Zhao, F. Cheng, Z. Yuan, S. Tian, X. Yang, J. Tian, Deep learning radiomics based on contrast-enhanced ultrasound images for assisted diagnosis of pancreatic ductal adenocarcinoma and chronic pancreatitis, BMC medicine 20(1) (2022) 74.

[59] W.-J. Tong, S.-H. Wu, M.-Q. Cheng, H. Huang, J.-Y. Liang, C.-Q. Li, H.-L. Guo, D.-N. He, Y.-H. Liu, H. Xiao, Integration of artificial intelligence decision aids to reduce workload and enhance efficiency in thyroid nodule management, JAMA network open 6(5) (2023) e2313674-e2313674.

[60] L. Wang, L. Chang, R. Luo, X. Cui, H. Liu, H. Wu, Y. Chen, Y. Zhang, C. Wu, F. Li, H. Liu, W. Guan, D. Wang, An artificial intelligence system using maximum intensity projection MR images facilitates classification of non-mass enhancement breast lesions, European Radiology 32(7) (2022) 4857-4867.

[61] Y. Wang, F. Liu, H. Zhang, Q. Wang, P. Yu, J. Wang, Z. Zhang, G. Wang, Y. Zhang, Y. Yang, Y. Mou, N. Mao, X. Song, Deep Learning Model for the Differential Diagnosis of Nasal Polyps and Inverted Papilloma by CT Images: A Multicenter Study, Academic Radiology 32(5) (2025) 2900-2909.

[62] L. Wan, X. Su, Z. Xiong, Z. Cui, G. Tang, H. Zhang, L. Zhang, Development and application of AI assisted automatic reconstruction of axial lumbar disc CT images and diagnosis of lumbar disc herniation, European Journal of Radiology 185 (2025) 112003.

[63] K. Wenderott, J. Krups, J.A. Luetkens, M. Weigl, Radiologists’ perspectives on the workflow integration of an artificial intelligence-based computer-aided detection system: A qualitative study, Applied ergonomics 117 (2024) 104243.

[64] T. Xia, S. Zhang, B. Zhao, Y. Lei, Z. Xiao, B. Chen, J. Zha, Y. Yu, Z. Wu, C. Lu, Attitudes of radiologists and interns toward the adoption of GPT-like technologies: a National Survey Study in China, Insights into Imaging 16(1) (2025) 30.

[65] Z. Xing, J. Chen, L. Pan, D. Huang, Y. Qiu, C. Sheng, Y. Zhang, Q. Wang, R. Cheng, W. Xing, Enhanced Detection of Prostate Cancer Lesions on Biparametric MRI Using Artificial Intelligence: A Multicenter, Fully-crossed, Multi-reader Multi-case Trial, Academic Radiology 32(10) (2025) 5954-5963.

[66] B. Yacoub, A. Varga-Szemes, U.J. Schoepf, I.M. Kabakus, D. Baruah, J.R. Burt, G.J. Aquino, A.K. Sullivan, J.O. Doherty, P. Hoelzer, Impact of artificial intelligence assistance on chest CT interpretation times: a prospective randomized study, American Journal of Roentgenology 219(5) (2022) 743-751.

[67] L. Yan, Z. Liang, H. Zhang, G. Zhang, W. Zheng, C. Han, D. Yu, H. Zhang, X. Xie, C. Liu, A domain knowledge-based interpretable deep learning system for improving clinical breast ultrasound diagnosis, Communications Medicine 4(1) (2024) 90.

[68] H. Yoo, E.Y. Kim, H. Kim, Y.R. Choi, M.Y. Kim, S.H. Hwang, Y.J. Kim, Y.J. Cho, K.N. Jin, Artificial Intelligence-Based Identification of Normal Chest Radiographs: A Simulation Study in a Multicenter Health Screening Cohort, Korean Journal of Radiology 23(10) (2022) 1009-1018.

[69] Y. Zhu, Z. Meng, X. Fan, Y. Duan, Y. Jia, T. Dong, Y. Wang, J. Song, J. Tian, K. Wang, Deep learning radiomics of dual-modality ultrasound images for hierarchical diagnosis of unexplained cervical lymphadenopathy, BMC medicine 20(1) (2022) 269.
